# Targeted RNA Knockdown by a Type III CRISPR-Cas Complex in Zebrafish

**DOI:** 10.1089/crispr.2020.0032

**Published:** 2020-08-24

**Authors:** Thomas Fricke, Dalia Smalakyte, Maciej Lapinski, Abhishek Pateria, Charles Weige, Michal Pastor, Agnieszka Kolano, Cecilia Winata, Virginijus Siksnys, Gintautas Tamulaitis, Matthias Bochtler

**Affiliations:** ^1^ International Institute of Molecular and Cell Biology, Warsaw, Poland.; ^2^ Institute of Biotechnology, Vilnius University, Vilnius, Lithuania.; ^3^ Polish Academy of Sciences, Institute of Biochemistry and Biophysics, Warsaw, Poland.

## Abstract

RNA interference is a powerful experimental tool for RNA knockdown, but not all organisms are amenable. Here, we provide a proof of principle demonstration that a type III Csm effector complex can be used for programmable mRNA transcript degradation in eukaryotes. In zebrafish, *Streptococcus thermophilus* Csm complex (StCsm) proved effective for knockdown of maternally expressed *EGFP* in germ cells of *Tg(ddx4:ddx4-EGFP)* fish. It also led to significant, albeit less drastic, fluorescence reduction at one day postfertilization in *Tg(myl7:GFP)* and *Tg*(*fli1:EGFP)* fish that express *EGFP* zygotically. StCsm targeted against the endogenous *tdgf1* elicited the characteristic one-eyed phenotype with greater than 50% penetrance, and hence with similar efficiency to morpholino-mediated knockdown. We conclude that Csm-mediated knockdown is very efficient for maternal transcripts and can also be used for mixed maternal/early zygotic and early zygotic transcripts, in some cases reaching comparable efficiency to morpholino-based knockdown without significant off-target effects.

## Introduction

Chronic knockout and acute knockdown of genes often lead to drastically different phenotypes, in ways that are only partly explained by technical limitations.^1,2^ Therefore, knockdown experiments remain interesting even in the era of facile DNA knockout generation using CRISPR-Cas9.

In many experimental animal models, systemic^3^ or cell-autonomous^4^ RNA knockdown can be achieved by exploiting endogenous RNA inference pathways, but there are important exceptions, zebrafish among them. In zebrafish embryos, endoribonuclease-prepared short interfering RNA reportedly leads to nonspecific developmental defects,^5^ presumably due to overload of the endogenous interference pathways. More encouraging results have been obtained using either short interfering RNA^5^ or short hairpin RNA,^6^ but neither method has gained widespread acceptance in the zebrafish community.

Morpholino-mediated knockdown is the leading technology for RNA silencing in zebrafish.^7–9^ The method is well established, but some limitations exist. Morpholinos only bind to RNA; but they do not cleave it. Therefore, only translation initiation and splice sites may be targeted, which is not always uniquely possible. Moreover, many maternal RNAs are already spliced and therefore very difficult to target. Morpholinos have a low “useful” range of concentrations. At lower concentrations, silencing is frequently incomplete,^10^ while at higher concentrations, off-target effects are common and have to be carefully controlled.^1,11^ Recently, dCas9-mediated transcriptional inhibition^12^ has been tested in zebrafish as an alternative to morpholinos, but success has only been demonstrated in a single case using relatively early embryos.^13^ For maternal transcripts, morpholino-based knockdown is problematic, and transcription suppression can altogether not be used.

Prokaryotic Argonaute proteins (Agos) provide an attractive, RNA degradation–based strategy for programmable, targeted knockdown. The approach becomes possible because some prokaryotic Agos, such as the *Marinitoga piezophila* Ago, differ in their guide nucleic acid requirements from endogenous Agos.^14^ *Natronobacterium gregoryi* Ago (NgAgo)—now considered as a DNA-guided, RNA-directed endonuclease^15^—is to our knowledge the only heterologous Ago that has been tested as a knockdown tool for zebrafish. In principle, NgAgo should make it possible to target maternal transcript as well or better than zygotic transcripts. However, to our knowledge, only knockdown of one early zygotic gene (*fabp11a*) has been tested, leading to eye development defects.^15^

RNA-directed CRISPR nucleases such as the class1 (multiple subunits) enzymes of the Csm (type III-A)^16,17^ and Cmr (type III-B)^18,19^ or class 2 (single subunit) effectors such as Cas13a (C2c2, type VI-A),^20–22^ Cas13b (type VI-B),^23^ and Cas13d (type VI-D)^24^ or Cas9 (type II) redirected to RNA using PAMmers^25^ could potentially be used for programmable RNA degradation. For Cas13 proteins, RNA knockdown in cells,^26,27^ plants,^28^ and animals, including zebrafish,^29^ has already been demonstrated. In this work, we have tested this concept using zebrafish as the animal model and type III-A Csm complexes as the programmable CRISPR RNA endoribonucleases.

The Csm proteins bound to CRISPR RNAs (crRNAs) form multisubunit protein–RNA complexes.^30^ Initial genetic data suggested that these complexes act as RNA-guided DNA endonucleases.^31^ However, subsequent *in vitro* studies indicated that the Csm complexes had RNA-guided, RNA-directed endoribonuclease activities cleaving substrate RNAs at multiple, regularly spaced sites.^16,17,32^ The apparent inconsistency between *in vivo* and *in vitro* data was resolved by the demonstration that the Csm complexes have transcription-dependent deoxyribonuclease (DNase) activity.^33^ Csm complexes find their targets by hybridization of the guide crRNA with the nascent RNA from transcription.^34^ They carry out co-transcriptional DNA and RNA cleavage during the expression phase of bacterial immunity, thus cleaving DNA and eliminating RNA of the invader.^32,34,35^

Active sites responsible for the DNase and ribonuclease (RNase) activities are distinct and located in different subunits of the Csm complex. The Cas10 subunit harbors an HD domain,^36^ which ensures the DNase activity of the Csm complex.^37^ Csm3 subunits, present in multiple copies and forming a crRNA binding filament structure, harbor the RNase activities of the Csm complex.^17^ Consistent with this assignment, the Cas10 D16A and Csm3 D33A mutations (*Streptococcus thermophilus* numbering) specifically abolish ssDNase and RNase activities of the complex, respectively.^17,34,35^ Csm complexes can so far only be assembled in bacterial cells. In the natural hosts, precursor crRNAs (pre-crRNAs) are expressed from CRISPR regions that are subjected to two cleavage events: primary processing by endoribonucleolytic cleavage of Cas6 to produce a ∼70 nt intermediate and further maturation during which non-Cas nucleases trim the 3′-end of the intermediates to generate matured ∼40nt crRNAs.^38,39^ The crRNA maturation and the loading on Csm complexes also occur in heterologous hosts carrying the Cas/Csm operon and associated CRISPR region.^17^

**FIG. 1.**
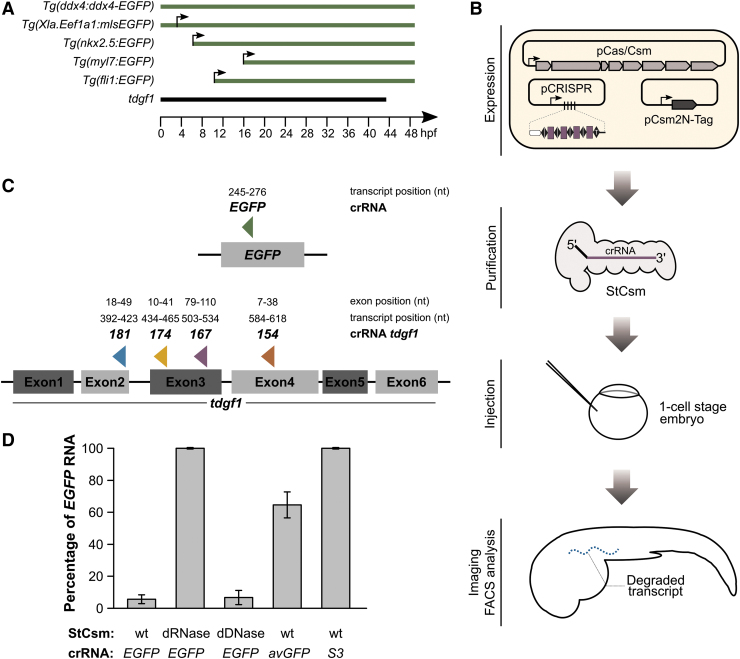
Experimental design and *in vitro* tool validation. **(A)** Onset of transcription of transcripts targeted in this study. The *Tg(ddx4:ddx4-EGFP* fish express the transgene strictly maternally, there is no zygotic expression for at least 50 hours postfertilization (hpf).^40^ In germ cells, the mRNA is exempt from the otherwise widespread RNA degradation at the mid-blastula transition.^55^ The *Tg(Xla.Eef1a1:mlsEGFP)* fish express *EGFP* with an N-terminal mitochondrial localization signal derived from subunit VIII of cytochrome oxidase under the control of the constitutive elongation factor 1α (EF-1α).^41^ Expression is both maternal and zygotic. The *Tg(nkx2.5:EGFP)*, *Tg(myl7:GFP)* and *Tg(fli1:EGFP)* express the transgene zygotically. The *Tg(nkx2.5:EGFP)* fish first exhibit fluorescence in the ventral margin of the embryo at the onset of gastrulation (∼5.5 hpf). By 2 days postfertilization (dpf), *EGFP* expression is limited to the heart.^42^ *Tg(myl7:GFP)* expression becomes detectable at ∼16 hpf in myocardial cells of the heart and persists for the lifetime of the animal.^43^ The *Tg(fli1:EGFP)* fish start to express *EGFP* from the three-somite stage (∼10 hpf). At 1 dpf, trunk and segmental vessels and cells of erythroid morphology are fluorescently labelled, and at 2 dpf, there is also fluorescence in the neural crest derived aortic arches and the developing jaw.^44^ Endogenous *tdgf1* is both maternally and zygotically expressed.^45^ **(B)** Experimental workflow. Cas/Csm proteins were co-expressed with a synthetic CRISPR array harboring four identical spacers (to produce only one type of StCsm complex), or four different spacers (to produce mixtures of StCsm complexes). Targeting and control StCsm complexes were purified as intact ribonucleoproteins from *Escherichia coli* extracts. Injections were done into the yolk of 1-cell stage embryos, and EGFP fluorescence, or phenotype was then monitored at later time points (1 dpf, 2 dpf, 5 dpf). **(C)** Location of the single site in the *EGFP* transcripts and of the four sites chosen for targeting in tdgf1 by our method. The names for *tdgf1* target sites and spacers (“181”, “174”, “176”, “154”) reflect our internal site scores. **(D)** Quantification of radioactively labeled target *EGFP* RNA after 1 h of *in vitro* incubation with different StCsm complexes. A low amount of leftover *EGFP* RNA indicates a high targeting efficiency.

For our knockdown experiments, we initially used zebrafish lines expressing enhanced green fluorescent protein (*EGFP*) from various promoters in different tissues and at different stages of development. The *Tg(ddx4:ddx4-EGFP),*^40^ *Tg(Xla.Eef1a1:mlsEGFP),*^41^ *Tg(nkx2.5:EGFP),*^42^ *Tg(myl7:GFP),*^43^ and *Tg*(*fli1:EGFP)*^44^ test cases were chosen to include cases of maternal RNA deposition without zygotic expression, maternal and zygotic expression, and zygotic expression only. In all cases, the *EGFP* transgenes were expressed from transposon insertion sites, always in the background of the endogenous genes (Fig. 1A).

Further, we tested knockdown efficiency on the endogenous target *teratocarcinoma-derived growth factor 1 (tdgf1*), previously known as *one-eyed pinhead*. The gene is maternally and zygotically expressed^45^ and zygotically required for the formation of ventral neuroectoderm, endoderm, and the prechordal plate.^46^ Because of the very clear knockdown phenotype characterized by defects in eye and somite development, the gene was previously used as a test case to monitor the efficacy of knockdown.^8^ In addition to the cyclopic phenotype, loss of *tdgf1* also leads to heart defects and edema.^47^

## Materials and Methods

Cas/Csm proteins were co-expressed from a synthetic CRISPR array containing four spacers, and StCsm(*EGFP*) and StCsm(*tdgf1*) complexes were purified essentially as previously described.^17^

*EGFP* and *tdgf1* knockdown experiments were done with a doses of 0.5 ng StCsm(*EGFP*) and 1.5 ng StCsm(*tdgf1*), respectively. For detailed methods, see the Supplementary Methods.

All sequencing data have been deposited in the Gene Expression Omnibus database (accession number GSE146852). Expression plasmids for synthetic CRISPR arrays and Csm subunits will be available from Addgene (see plasmid list in Supplementary Table S1).

## Results

### Study design

For the zebrafish RNA knockdown experiments, we chose the well characterized type III-A CRISPR-Cas effector Csm complex from *S. thermophilus* (StCsm)^17,34,39,48^ as our model type III CRISPR endonuclease. The Cas/Csm proteins coding genes were co-expressed with a synthetic CRISPR array (Fig. 1B), which contained either four identical copies of a spacer (to make one type of complex only) or four different spacers (to make a mixture of different complexes). For the *EGFP* targeting experiments, the targeted region was chosen arbitrarily in the coding region, and a CRISPR array with only one type of spacer was used. For the *tdgf1* knockdown experiments, four targeting sites were chosen. As our method targets mRNAs in the cytoplasm that are expected to be spliced already, targeting regions had to be placed in exons. We focused on coding regions and gave preference to regions with balanced GC-content regions with low probability for secondary structure formation, avoiding known polymorphisms, and avoiding regions that are similar to regions elsewhere in the genome (to minimize off-target effects). Two types of CRISPR arrays were prepared, one with only a single type of spacer (termed “167”), and another one with four different spacers (termed “154,” “167,” “174,” and “181”) (Fig. 1C).

The Cas/Csm proteins together with synthetic CRISPR arrays were co-expressed and assembled into StCsm complex in heterologous *Escherichia coli* host. The intact ribonucleoprotein particles were then pulled down from the *E. coli* extracts using a tagged Csm2 subunit as a bait by subsequent Strep-chelating affinity and size exclusion chromatography. Purified StCsm complexes were injected into the yolk of one-cell stage zebrafish embryos. Apart from wildtype (wt) StCsm complexes, we used complexes containing the DNase-deficient Cas10 D16A variant (henceforth termed defective DNase or dDNase StCsm) or the RNase-deficient Csm3 D33A variant (henceforth termed defective RNase or dRNase StCsm). For *EGFP* transcript knockdown we also used StCsm(*avGFP*) which contains imperfectly complementary crRNA, and StCsm(*S3*) which contains a completely unrelated crRNA guide^17^ (Supplementary Table S2). For the *EGFP* target, we then monitored EGFP fluorescence until 5 days postfertilization (5 dpf) and quantified this fluorescence by fluorescence-activated cell sorting (FACS) at 1 dpf and 2 dpf. For the *tdgf1* target, phenotypes were scored at 1 dpf (Fig. 1C).

### Validation of the RNase and DNase activities of StCsm complexes

Prior to their use in animals, the composition and activities of StCsm complexes were tested. For wt and mutant StCsm(*EGFP*) complexes, Coomassie staining demonstrated a good purity and a protein subunit composition compatible with the previous results^17^ (Supplementary Fig. S1A). SYBR Gold staining showed that the all StCsm complexes contained a mixture of crRNAs (Supplementary Fig. S1B) which resulted after partly processing of pre-crRNA by *E. coli* host enzymes as described earlier.^17,39^ A 72-nt long crRNA raised from processing of the pre-crRNA, and 40-nt long crRNA raised from further trimming at the final maturation stage of crRNA.

Further, RNase activity of StCsm complexes was monitored at 28°C, the temperature used for the zebrafish experiments. Time courses were recorded, and the amount of RNA target after fixed interval of incubation was quantified. The results confirm the on-target RNase activity of wt StCsm and dDNase StCsm, but not of dRNase StCsm, when a fully target-complementary crRNA guide is used. The results also demonstrate that even a few mismatches (here *avGFP* versus *EGFP*) suffice to drastically reduce the RNase activity, to levels not much higher than observed with a completely unrelated crRNA guide (Fig. 1D and Supplementary Fig. S2A and B). DNA hydrolysis experiments confirmed that purified wt and dRNase StCsm complexes were also effective for cleaving single stranded DNA in the presence of target RNA (Supplementary Figs. S2C and S2D). However, since StCsm complexes were only targeted to the cytoplasm and not the nucleus in our experiment, the DNase activity was not expected to be relevant for *in vivo* efficacy. *In vitro* testing results for StCsm(*tdgf1*) complexes were analogous to those for StCsm(*EGFP*) (Supplementary Fig. S3).

### Knockdown of the maternal *EGFP* in *Tg(ddx4:ddx4-EGFP)*

The endogenous ddx4(*vasa*) gene and *EGFP* in the *Tg(ddx4:ddx4-EGFP)* transgenic line are maternally expressed.^49^ Literature reports indicate that embryonic transcription sets in at the gastrulation stage for the endogenous transcript, and even later for the *EGFP* transgene.^40,50^ In our hands, offspring from *Tg(ddx4:ddx4-EGFP)* positive fathers and wt mothers remained nonfluorescent for the first five days after fertilization (monitoring was ended at this time because of legal restrictions), confirming the *EGFP* expression as strictly maternal (Supplementary Fig. S4), and therefore a relatively “easy” target for StCsm(*EGFP*)-controlled RNA knockdown.

In preliminary experiments, we tested potential toxicity and efficacy of StCsm injections for various injection volumes and complex concentrations. To limit mechanical damage to the embryos, injections were made to the yolk, and not the embryo proper. Injection volumes of 1 nL and below were well tolerated, up to an StCsm(*EGFP*) concentration of 2.5 mg/mL (the highest concentration that we could test with our StCsm complex master stocks) (Supplementary Fig. S5A). Further effects showed that injections of 1 nL of 0.5 mg/mL StCsm(*EGFP*) complex were sufficient for knockdown (Supplementary Fig. S5B). In the following, we describe only the injection experiments with this dose.

**FIG. 2.**
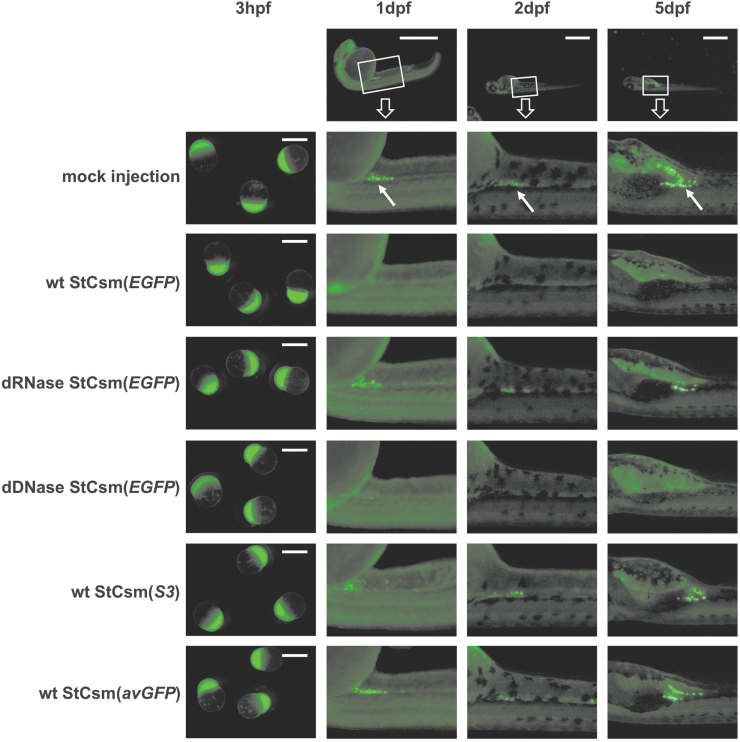
Microscopy of StCsm mediated *EGFP* knockdown in *Tg(ddx4:ddx4-EGFP)* fish. Fluorescence from *Tg(ddx4:ddx4-EGFP)* fish after injection with wildtype (wt) or mutant StCsm complexes are shown. The arrows indicate the location of primordial germ cells. Injection was done at the 1-cell embryo stage, observations were at 3 hpf, 1 dpf, 2 dpf, and 5 dpf. The scale bar represents 1 mm. Boxes in the top row show the region of the embryo magnified in panels below. Arrows point to germ cells.

The use of 0.5 ng StCsm(*EGFP*) crRNA did not reduce EGFP fluorescence in the early stages of development (3 hours postfertilization [hpf]), presumably due to maternally deposited protein. By 1 dpf, similar weak background (primarily in the brain) was seen in both injected embryos and noninjected controls, but there was a clear difference in germ cells. Fluorescence was strong in germ cells of control embryos, but almost completely extinguished in germ cells of StCsm(*EGFP*) injected embryos. The loss of fluorescence in germ cells (compared with control) persisted during the entire observation period ending at 5 dpf (Fig. 2). Knockdown was dependent on the StCsm(*EGFP*) RNase, but not on DNase activity. The DNase-deficient mutant dDNase StCsm(*EGFP*) extinguished fluorescence at 1 dpf like the wt complex, whereas the RNase-deficient StCsm(*EGFP*) failed to do so. Poor or absent complementarity of the crRNA to its target also abolished the knockdown. Both StCsm(*avGFP*) and StCsm(*S3*) did not reduce EGFP germ cell fluorescence (Fig. 2).

**FIG. 3.**
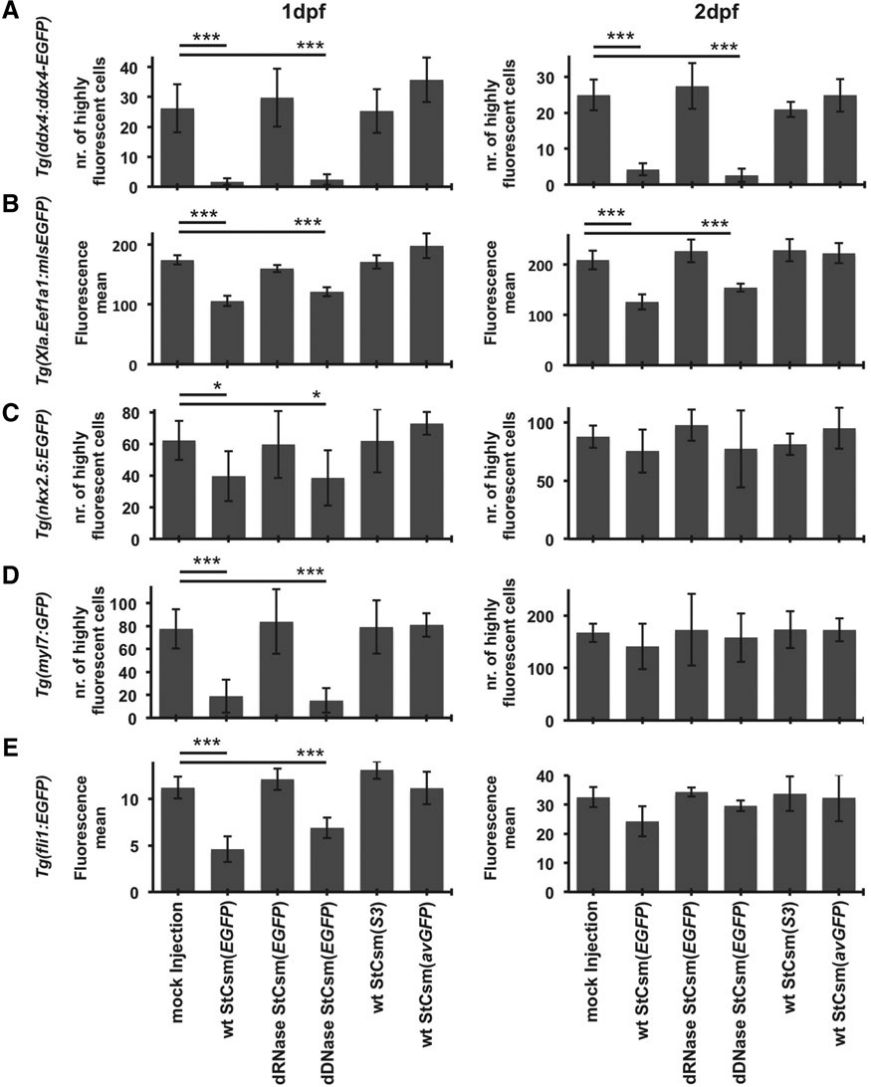
Quantification of *EGFP* knockdown efficiency by fluorescence-activated cell sorting analysis. Enhanced green fluorescent protein fluorescence in **(A)** *Tg(ddx4:ddx4-EGFP)*, **(B)** *Tg(Xla.Eef1a1:mlsEGFP)*, **(C)** *Tg(nkx2.5:EGFP)*, **(D)** *Tg(myl7:GFP)* and **(E)** *Tg(fli1:EGFP)* fish was quantified 1 dpf and 2 dpf by flow cytometry of minced and trypsinized embryos. For *Tg(ddx4:ddx4-EGFP)*, *Tg(nkx2.5:EGFP)* and *Tg(myl7:GFP)* embryos, the number of highly fluorescent cells (at least 50-fold background fluorescence) was counted. Results are from 3 independent experiments; error bars represent one standard deviation. *P < 0.05 as compared with respective controls, ***P < 0.001 as compared with respective controls. Data are represented as mean ± SEM.

To make the above observations quantitative, we minced and trypsinized embryos from the mating of a single pair of fish and subjected the resulting pool of cells (and aggregates of cells) to FACS. In order to avoid the influence of background fluorescence, only highly fluorescent cells (50-fold higher fluorescence than mean) were counted. Almost all of these cells exhibited strong side scatter that would only be expected from large cells such as primordial germ cells,^51^ confirming that germ cells were indeed gated. At 1 dpf and 2 dpf, high-EGFP cells were 20-fold and 6-fold less frequent respectively in both wt and dDNase StCsm(*EGFP*)-injected fish than in controls. Fluorescence counts were not altered when the dRNase StCsm(*EGFP*), the wt StCsm(*avGFP*) complex with imperfectly complementary crRNA, or the StCsm(*S3*) complex with target-unrelated crRNA were used (Fig. 3A).

### Knockdown of the maternal-zygotic *EGFP* in Tg(Xla.Eef1a1:mlsEGFP)

Embryos from *Tg(Xla.Eef1a1:mlsEGFP)* show green fluorescence in mitochondria already in oocytes, and throughout the life of the fish. In crosses between wt ABTL fish and *Tg(Xla.Eef1a1:mlsEGFP)* fish, fluorescence is observed throughout embryonic development when the mother carries the *Tg(Xla.Eef1a1:mlsEGFP)* transgene, indicating maternal and then zygotic expression, whereas fluorescence is observed only after the midblastula transition in the reciprocal cross (Supplementary Fig. S6A). For knockdown experiments, we used the same concentrations of StCsm(*EGFP*) and variants as in the *Tg(ddx4:ddx4-EGFP)* experiment. The lines were analyzed by fluorescence microscopy and FACS analysis of digested embryos at 1 dpf, and in the case of the *Tg(Xla.Eef1a1:mlsEGFP)* incross, also at 2 dpf. As green fluorescence is present throughput the embryo and not concentrated in a particular cell type, we quantified mean fluorescence, instead of the number of highly fluorescent cells as for the *Tg(ddx4:ddx4-EGFP)* fish.

When we used fish from *Tg(Xla.Eef1a1:mlsEGFP)* fathers and wt mothers, knockdown efficiency using wt StCsm(*EGFP*) was quite good. The knockdown efficiency was insignificantly lower with the dDNase variant, arguing for a possible contribution of DNA cleavage, perhaps during the frequent M-phases, in the observed reduction of fluorescence. However, the dRNase variant, which has only DNase activity, was completely ineffective, suggesting that the reduction of fluorescence was not due StCsm(*EGFP*) DNase activity (Supplementary Fig. S6B). In the reciprocal cross, StCsm(*EGFP*) injection reduces fluorescence to a much lesser extent, either because maternal RNA overwhelms StCsm(*EGFP*) capacity, or more likely, because fluorescence of maternally deposited EGFP protein is not affected by the knockdown (Supplementary Fig. S6B). A similarly low knockdown efficiency was also observed when the StCsm(*EGFP*) was pitched against maternally deposited RNA and zygotically expressed RNA in embryos from crosses of *Tg(Xla.Eef1a1:mlsEGFP)* parents (Fig. 3B).

### Knockdown of the zygotic *Tg(myl7:GFP)* and *Tg(fli1:EGFP)*, but not *Tg(nkx2.5:EGFP)*

To confirm that StCsm(*EGFP*) could also be used for knockdown of zygotically expressed mRNAs, we tested knockdown of *EGFP* transcripts from other promoters. Consistent with predominantly zygotic promoter activity, heterozygotic *Tg(nkx2.5:EGFP)*, *Tg(myl7:GFP)* or *Tg*(*fli1:EGFP)* fish carrying a maternally or paternally inherited transgene exhibited similar fluorescence at all tested times (4 hpf, 1 dpf, 2 dpf, and 5 dpf). As before, the amount of StCsm(*EGFP*) for injections was not optimized anew, and the same concentration as for the *Tg(ddx4:ddx4-EGFP)* experiments was used throughout. All lines were analyzed by fluorescence microscopy and FACS analysis of digested embryos at 1 dpf and 2 dpf.

The *Tg(nkx2.5:EGFP)* and *Tg(myl7:GFP)* fish exhibit fluorescence in the heart. We quantified knockdown efficiency as for the *Tg(ddx4:ddx4-EGFP)* line by counting the number of highly fluorescent cells (50-fold more fluorescent than background). A knockdown of 30%–40% was detected in the *Tg(nkx2.5:EGFP)* fish upon StCsm(*EGFP*) injection by FACS (Fig. 3C), which was not detectable by imaging. A stronger, 4-fold fluorescence reduction could be achieved in the *Tg(myl7:GFP)* line at 1 dpf (Fig. 3D and Supplementary Fig. S7). Controls with the dDNase and dRNase StCsm(*EGFP*) variants showed that the reduction of fluorescence was almost exclusively due to the RNase activity of the StCsm(*EGFP*) complex in both cases. At 2 dpf, the effect of knockdown had essentially faded away.

The *Tg*(*fli1:EGFP)* fish exhibit fluorescent vasculature throughout the embryo. Therefore, we quantified knockdown efficacy by mean fluorescence in this case. At 1 dpf, more than 50% knockdown was achieved using the wt StCsm(*EGFP*) complex. The reduction of EGFP fluorescence was slightly lower with the dDNase variant, raising the possibility that the DNase activity of the StCsm complex may have contributed the reduction of fluorescence. However, the RNase-deficient version of the complex was ineffective, demonstrating that the RNase activity is vital for StCsm-generated knockdown. At 2 dpf, knockdown effects were reduced to statistically insignificant levels (Fig. 3E and Supplementary Fig. S8).

### Targeting of the endogenous *tdgf1* transcript using StCsm complex

The *tdgf1* gene has been used as a test case for morpholino-based knockdown because of easily scored and rather characteristic one-eyed pinhead phenotype that is observed in optimal conditions at 1 dpf.^8^ In most experiments, however, a spectrum of phenotypes is observed. We classified the phenotype as mild to moderate (defects in eye development, leading to reduced size of one of the eyes, and/or defects in the notochord and tail region), strong (cyclopic phenotype characterized by the presence of a single eye, or fused eyes, with defects in the notochord region), and severe (reduced head region, often with single eye significantly reduced in size, and edema, defects in general body axis, notochord and tail region) (Supplementary Fig. S9). Especially for the latter phenotype, the boundary between “desirable” on-target and “undesirable” off-target effects is still not fully clear.

**FIG. 4.**
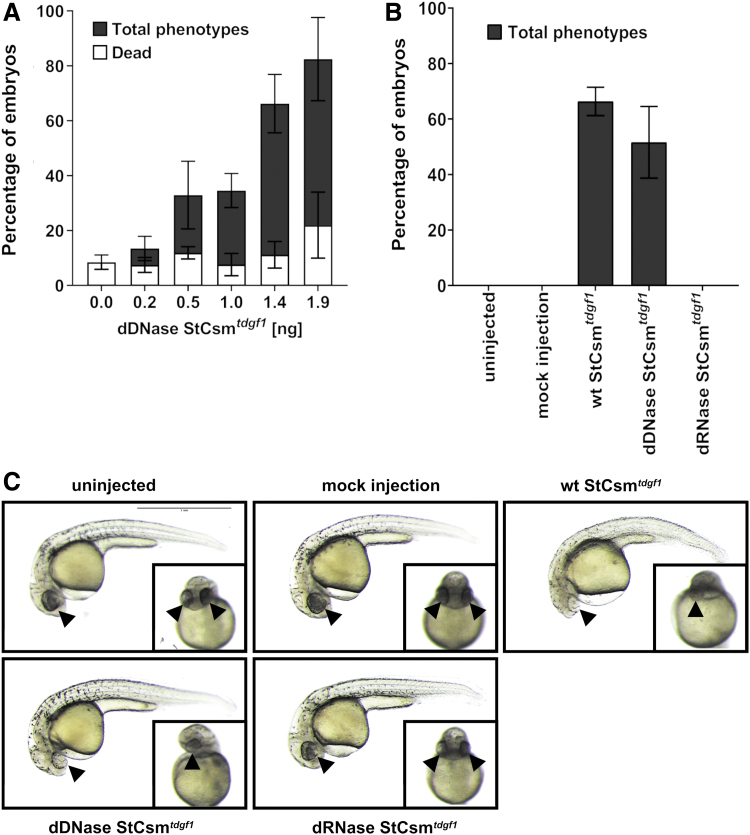
Knockdown of endogenous *tdgf1*. **(A)** Dose-dependent penetrance and lethality were assessed after injection of dDNase StCsm(*tdgf1*^167,174,154,181^). Different amounts of dDNase StCsm(*tdgf1*^167,174,154,181^) were injected in a constant volume of 1 nL at the 1-cell stage. Phenotypes were screened after 24 hpf on the basis of morphological changes. Data represented as mean ± SEM. **(B)** Bar plot comparing the efficiency of different StCsm complexes. 1.5 ng of either wt, dRNase or dDNase StCsm(*tdgf1*^167,174,154,181^) complexes in 1 nL volume was injected in the same clutch of embryos. Results from injections in five different clutches of embryos are presented. Data represented as mean ± SEM. **(C)** Lateral and ventral images of the injected embryos after 24 hpf. Arrows indicate position of eye or eyes. Scale bar represents 1 mm.

Injections of the single guide wt StCsm(*tdgf1*^167^) elicited only mild to moderate, but not strong or severe, phenotypes. A mixture of wt StCsm complexes with four different guides (*tdgf1*^167,174,154,181^) proved more effective. To get the optimal effects, we carried out a dose–response study for 1 nL injection volume and variable ribonucleoprotein concentration. When ∼0.25 ng was injected, lethality was very low at around 10%, comparable to the lethality associated with mock injections, but mostly mild and moderate phenotypes were observed, and total penetrance was low. Penetrance (for all phenotypes, from mild to severe) increased with the ribonucleoprotein dose and reached over 50% for ∼1.4 ng injections, essentially without increasing mortality (Fig. 4A). Increasing injection dose yet further was not helpful. Penetrance did not increase much, but lethality increased considerably.

As the *tdgf1* phenotype was scored at 1 dpf, long after the onset of zygotic transcription at 3.5 hpf, attribution of the phenotype to RNA knockdown, rather than DNase-directed endonuclease activity relied on the expected cytoplasmic localization of the complex only. In order to confirm that the phenotype was indeed attributable to RNA knockdown, we compared the efficacy of wt StCsm(*tdgf1*^167,174,154,181^) with the similarly loaded dDNase and dRNase variants at the same concentration (Fig. 4B). As anticipated, phenotypes were observed only if RNase activity was intact. The penetrance of phenotypes was only slightly (about 5%) lower for the DNase-deficient variant than for wt complex. Moreover, phenotypes were observed in very similar proportions. Therefore, we conclude that all or nearly all of the phenotype was attributable to RNA degradation and was not due to mutations induced by imprecise repair of induced double-strand breaks in DNA (Fig. 4C).

### Quantification of on- and off-target effects for *EGFP* knockdown by RNA-Seq

For quantification of on- and off-target knockdown effects, *Tg(ddx4:ddx4-EGFP)* females were crossed with *casper* males. Offspring of three mating pairs were pooled at the time of collection and randomly divided into injection and mock injection (control) groups. Injection dose was 0.7 ng (in 1 nL), and therefore very similar to the dose used in earlier experiments (Figs. 2 and 3). For the injected embryos, 25 each were harvested, pooled, and snap frozen in liquid nitrogen at the 128-cell stage, at 5 hpf, and at 24 hpf. The same was also done for control embryos and all the injected samples were stored in the -80°C freezer until further processing. Three independent experiments were performed, and RNA was isolated by polyT-polyA hybridization limiting detection to full-length RNAs and 3′ fragements. In total, we obtained and analyzed RNA-Seq data for injected and control embryos, for three time points, in triplicate.

**FIG. 5.**
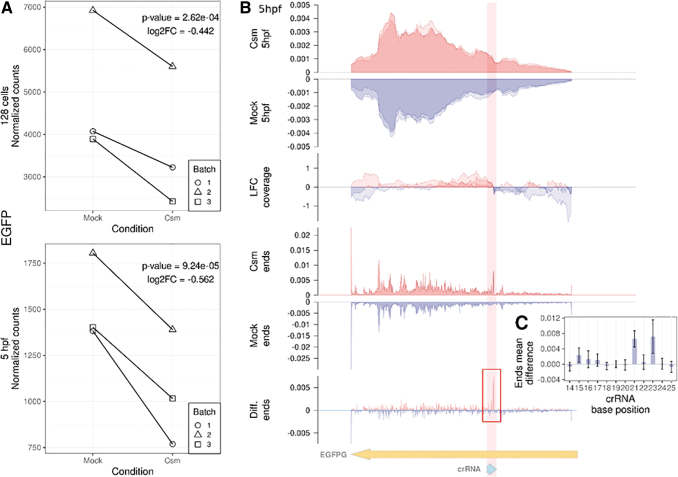
Analysis of on-target effects of *EGFP* knockdown. **(A)** Changes in *EGFP* read counts at the 128-cell stage (top panel) and 5 hours post fertilization (bottom panel). Black lines connect data points from the same replica batch. Each plot is annotated with log2 fold change value and *P*-value from the likelihood-ratio test. **(B)** Read coverage analysis of the *EGFP* transcript at 5 hpf. The top two panels (StCsm 5 hpf and mock 5 hpf) depict the read coverage represented as a fraction of total coverage at each position for the StCsm injected (red) and mock injected (blue) samples. Each replicate is plotted independently with the color shades gaining intensity where the plots overlap. The third panel (log-fold change [LFC] coverage) shows the log2 of the ratio between the read coverage of the StCsm injected and mock injected samples (positive values red, negative values blue). The fourth and fifth panel (StCsm ends and Mock ends) depicts the distribution of sequencing fragment ends over the whole *EGFP* transcript sequence for the StCsm injected (red) and mock injected (blue) samples. The sixth panel (Diff. ends), shows the difference in the sequencing fragment end distribution between the two samples, obtained by subtracting the mean amount of fragment ends of the mock sample from the mean amount of fragment ends at each coordinate of the StCsm sample. The positive values are colored red and negative are blue. The red rectangle illustrates the space enlarged on panel (C) in relation to the CRISPR RNA (crRNA) sequence. **(C)** The difference in the sequencing fragments end means at each coordinate in relation to the crRNA sequence. Error bars show 95% confidence interval.

We first focused on the on-target effects of StCsm(*EGFP*). As *EGFP* expression varied strongly between groups (we had both homo- and heterozygous fish), batch effect was added to the underlying models. Within group comparisons showed clear reduction of *EGFP* reads (Fig. 5A). A likelihood ratio test confirmed that a model with injection status (true or mock injection) modelled *EGFP* abundance significantly better than a model distinguishing only batches, for both the 128-cell stage and at 5 hpf (*P* = 3e-4 for 128-cell stage; *P* = 1e-4 for 5hpf). Meaningful comparisons were not possible for the 24 hpf samples, because *EGFP* read counts were too low, despite the persistence of fluorescence in germ cells.

Next, we compared the distributions of reads along the *EGFP* transcript for injected and mock injected embryos. At 128-cell stage and 5 hpf, the two distributions were broadly similar, as would be expected for complete transcript removal by endogenous RNA degradation pathways after the initial Csm cleavage (Fig. 5B, top, and Supplementary Fig. S10). For more detailed comparisons, we determined the logarithm of the ratio of reads in the injected and mock injected samples (log-fold coverage [LFC]). This analysis revealed that in the injected group, reads were underrepresented on the 5′-side and overrepresented on the 3′-side of the expected cleavage site in the *EGFP* transcript (notice that for the *EGFP* transcript in Fig. 5B and Supplementary Fig. S9, 5′ is on the right and 3′ on the left). We attribute the underrepresentation of reads on the 5′-side in injected embryos to the loss of 5′-fragments after cleavage; the overrepresentation of reads on the 3′-side can be understood as an effect of overall read number normalization (Fig. 5B and Supplementary Fig. S10, “LFC coverage”). Hence, the data show that the 3′-fragements of some *EGFP* transcripts persist after StCsm(*EGFP*) mediated cleavage. This conclusion was also supported by a mapping of read ends. Read ends are distributed very irregularly, but the distribution is very similar for injected and mock injected embryos. The exception to this rule is the predicted cleavage site, where read ends peak in the injected (Fig. 5B, “Csm ends,” and Supplementary Fig. S10), but not the mock injected sample (Fig. 5B, “Mock ends,” and Supplementary Fig. S10). As StCsm complexes can catalyze multiple cuts in their targets with a 6-nt stagger,^17^ we looked at higher resolution for this pattern, but did not see evidence for cleavage sites six nucleotides apart (Fig. 5C).

Although crRNA should not be enriched in our polyA-polyT hybridization based protocol and should therefore not be represented in our stranded library, a clear peak on the *EGFP* complementary strand was observed in the binding region of crRNA (not shown). These reads, attributed to crRNA contamination due to nonspecific bead binding, provided an opportunity to estimate crRNA persistence in the embryo. Averaged over the three replicas, the fraction of crRNA compared to total polyA-tailed RNA goes down by less than a factor of three between the 128-cell stage and 24 hpf.

**FIG. 6.**
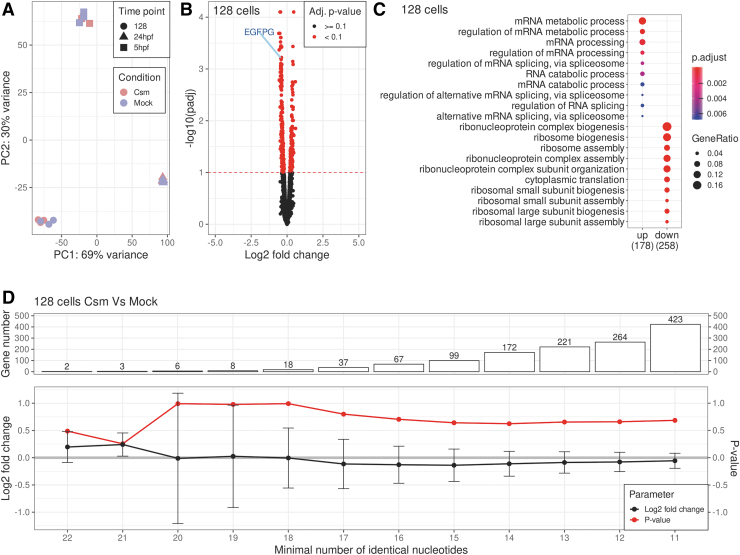
Analysis of off-target effects of *EGFP* knockdown. **(A)** Principle component analysis of mRNA expression levels in all sequenced samples. The first two principle components are shown and are annotated by the percentage of the variance explained by each of those components. The colors differentiate the samples by experimental condition, while the shape by analysis time point. **(B)** Volcano plot showing the relation of the log2 transformed fold changes of read counts at 128-cell stage to their log10 transformed *P*-values adjusted for multiple comparison tests with the Benjamini and Hochberg correction, as implemented in DESeq2. The point originating from the *EGFP* transcript has been marked. The dashed red line shows the 0.1 adjusted p-value threshold. **(C)** Comparison of biological process gene ontology terms enrichment of the upregulated and downregulated genes. Dots represent the term enrichment with color coding: red indicates high enrichment and blue indicates low. The sizes of the dots represent the ratio of genes containing the relevant term within up or down-regulated groups to the total number of genes in that group. **(D)** The plots showing the log2 transformed fold change and its respective *P*-value from likelihood ratio test for the full model, containing both condition and batch factors, and the reduced model containing only the batch, fitted to the counts of the groups of genes sharing the same minimal number of complementary nucleotides to the crRNA. The error bars show the standard error of the estimate. With each required nucleotide less, new genes are added to the tested group. The bar plot in the uppermost panel depicts the size of the relevant group.

Having confirmed the expected on-target effects of the StCsm complexes, we next focused on potential off-target effects. For each condition and repeat, at least 10 million assigned read counts were available. Scatter plots and Pearson correlation coefficients consistently above 0.9 showed that transcript abundances for the replica datasets were very consistent internally (correlation >0.9 for any replica pair). Principle component analysis (Fig. 6A) and hierarchical clustering (Supplementary Fig. S11A) grouped transcriptomes according to developmental stage. Transcript abundances were very similar for injected and noninjected embryos at any given stage (Supplementary Fig. S11B to D). Volcano plots indicated that at any given stage, few transcripts varied significantly in abundance (Fig. 6B for the 128-cell stage). *EGFP* itself, treated here like an endogenous gene, was among the highest scorers for differential expression overall, and for under-expression in the StCsm(*EGFP*) injected samples. Differentially expressed genes of course do not need to be direct StCsm(*EGFP*) targets.

To better understand the pattern of expression changes, we mapped the differentially expressed genes to GO categories. The top categories in this list are mostly related to metabolism, splicing, and protein expression. Overall, the GO pattern is very consistent with the idea that StCsm(*EGFP*) complexes alter the transcriptome primarily by causing a developmental delay (Fig. 6C). By contrast, distant sequence similarity between the crRNA exact target in *EGFP* and potential off-target effects in other transcripts did not seem to matter for regulation. As any downregulation was not significant on the level of single genes, we grouped transcripts according to the number of paired bases between them and crRNA. Spacers are 32 nt long. The two most similar genes could pair with the spacer region of the crRNA in 22 positions, or with 10 mismatches. The more mismatches were allowed, the larger the number of potentially affected genes. However, no major expression change was observed for all groups, and in particular, there was no trend that groups of genes with better complementarity to the crRNA were more downregulated than groups with worse complementarity (Fig. 6D and Supplementary Fig. S11E).

We conclude from this analysis that all transcripts are too far away from complementarity to be directly downregulated. This finding is in line with the observation that StCsm(*avGFP*), which is mismatched in six positions against the *EGFP* transcript, does not degrade this transcript (Fig. 1D).

## Discussion

Our experiments with StCsm variants show that reductions in *EGFP* fluorescence are largely independent of the DNase activity of the Csm complex, even for *EGFP* expressed from zygotically active promoters. This could reflect faster RNA than DNA degradation in part due to transcriptional silence up to the maternal to zygotic transition (MZT), or the exclusion of the StCsm complex from the nucleus due its large size and the absence of a nuclear localization signal (NLS). It remains to be tested how nuclear targeting of mRNAs or pre-mRNAs by StCsms with NLS compares with cytoplasmic targeting. Although we did not see evidence for DNase activity of the StCsm complex in our experiments, we recommend using the “cleaner” Csm dDNase variant for RNA knockdown experiments.

StCsm-mediated RNA knockdown worked best for the exclusively maternal *EGFP* transcript generated under the control of the *ddx4* promoter. The knockdown for transcripts expressed in the zygote was less pronounced than for the maternally deposited RNA, and the effect decreased over time. Observation of significant knockdown effects for the *myl7* and *fli1* promoter driven *EGFP* transcripts strongly suggests that the StCsm bound crRNA survives the widespread RNA degradation during the MZT at 3.5 hpf, most likely due to protection of the crRNA along its entire length by the StCsm complex.^17^ At 1 dpf, *EGFP* fluorescence was reduced less than two-fold in the *Tg(Xla.Eef1a1:mlsEGFP)* and *Tg(nkx2.5:EGFP)* lines. As most vertebrate genes are haplo-sufficient,^52,53^ this efficiency may not be enough to elicit phenotypes. Therefore, for now we recommend Csm-mediated knockdown only for maternally deposited RNAs and for use very early in development (at most 2 dpf).

We regard this work as a proof of principle for type III CRISPR-Cas based RNA knockdown in animals. Several potential avenues to increase the efficiency of targeting, and to make the method amenable to targets expressed later in development, remain to be explored. We have so far only tested the Csm from one bacterial species, and even with this complex, there may be room for improvement. It is possible that targeting pre-mRNAs and RNAs at the source in the nucleus (using a Csm DNase-deficient complex with NLS) could be more effective than cytoplasmic targeting for zygotic transcripts, because Csm complex would more stable against degradation by proteases. We have used pre-formed StCsm complexes. Assembled StCsm complexes from injected RNAs or RNAs made by transcription in zebrafish from DNA templates could be used alternatively. Finally, we have injected into the yolk only, to keep lethality low, and have not yet compared efficiencies for injection into yolk and embryo proper.

With maternal RNA transcripts and early zygotic transcripts within its scope, Csm knockdown currently fills a niche for knockdown in zebrafish that is not well addressed by the morpholino approach and inaccessible to the dCas9 transcription suppression approach. As zygotic transcription sets in late in zebrafish, many important biological questions, including questions of cell fate, RNA translation control, and others, can be addressed during this window.

The main competitor for Csm-based knockdown is Cas13-based knockdown. Initial successes have been reported in a preprint using Cas13.^29^ Those Cas13 experiments have so far been done with co-injection of mRNA for Cas13 and crRNA. In this experimental setup, the endonuclease first has to be synthesized, and must find its crRNA guide in the zebrafish embryo, before it can be active. On the other hand, the presence of mRNA means that endonuclease can be resupplied, perhaps increasing knockdown persistence. Our preliminary experiments indicate that it is also effective to co-inject Cas13 protein and crRNA. Compared to the Csm method, the Cas13-based knockdown could prove more amenable to parallel experiments, since the endonuclease can be loaded with crRNA *in vitro*. This is still difficult for Csm complexes, which have to be assembled in the producer organism, so that separate purifications are necessary if multiple targets have to be tested. On the other hand, Cas13 proteins have been reported to acquire nonspecific endonuclease activity, once they have cleaved a specific target,^54^ which could represent an off-target concern. Finally, the bacterial Argonaute protein NgAgo, now believed to a DNA guided RNase, has also been used for RNA knockdown, although so far only for one example, the *fabp11a* gene.^15^ The use of heterologous nucleic acid guided endonucleases to target RNA is clearly promising: direct comparisons are now required to reveal the relative merits of the methods that have now been shown to work at least in principle.

## Note in Proof

While this manuscript was in production, an amended version of reference 29 was published as Kushawah, G. et al., CRISPR-Cas13d Induces Efficient mRNA Knockdown in Animal Embryos, https://doi.org/10.1016/j.devcel.2020.07.013

## Supplementary Material

Supplemental data

Supplemental data

Supplemental data

Supplemental data

Supplemental data

Supplemental data

Supplemental data

Supplemental data

Supplemental data

Supplemental data

Supplemental data

Supplemental data

Supplemental data

Supplemental data

## References

[1] Kok FO, Shin M, Ni CW, et al. Reverse genetic screening reveals poor correlation between morpholino-induced and mutant phenotypes in zebrafish. Dev Cell. 2015;32:97–108. DOI: 10.1016/j.devcel.2014.11.018 25533206 PMC4487878

[2] Rossi A, Kontarakis Z, Gerri C, et al. Genetic compensation induced by deleterious mutations but not gene knockdowns. Nature. 2015;524:230–233. DOI: 10.1038/nature14580 26168398

[3] Tabara H, Grishok A, Mello CC RNAi in *C. elegans*: soaking in the genome sequence. Science. 1998;282:430–431. DOI: 10.1126/science.282.5388.430 9841401

[4] Dietzl G, Chen D, Schnorrer F, et al. A genome-wide transgenic RNAi library for conditional gene inactivation in *Drosophila*. Nature. 2007;448:151–156. DOI: 10.1038/nature05954 17625558

[5] Liu W-Y, Wang Y, Sun Y-H, et al. Efficient RNA interference in zebrafish embryos using siRNA synthesized with SP6 RNA polymerase. Dev Growth Differ. 2005;47:323–331. DOI: 10.1111/j.1440-169X.2005.00807.x 16026540

[6] De Rienzo G, Gutzman JH, Sive H Efficient shRNA-mediated inhibition of gene expression in zebrafish. Zebrafish. 2012;9:97–107. DOI: 10.1089/zeb.2012.0770 22788660 PMC3444767

[7] Bill BR, Petzold AM, Clark KJ, et al. A primer for morpholino use in zebrafish. Zebrafish. 2009;6:69–77. DOI: 10.1089/zeb.2008.0555 19374550 PMC2776066

[8] Nasevicius A, Ekker SC Effective targeted gene 'knockdown' in zebrafish. Nat Genet. 2000;26:216–220. DOI: 10.1038/79951 11017081

[9] Heasman J Morpholino oligos: making sense of antisense? Dev Biol. 2002;243:209–214. DOI: 10.1006/dbio.2001.0565 11884031

[10] Eisen JS, Smith JC Controlling morpholino experiments: don't stop making antisense. Development. 2008;135:1735–1743. DOI: 10.1242/dev.001115 18403413

[11] Stainier DY, Kontarakis Z, Rossi A Making sense of anti-sense data. Dev Cell. 2015;32:7–8. DOI: 10.1016/j.devcel.2014.12.012 25584794

[12] Shalem O, Sanjana NE, Zhang F High-throughput functional genomics using CRISPR-Cas9. Nat Rev Genet. 2015;16:299–311. DOI: 10.1038/nrg3899 25854182 PMC4503232

[13] Dong X, Li J, He L, et al. Zebrafish Znfl1 proteins control the expression of hoxb1b gene in the posterior neuroectoderm by acting upstream of pou5f3 and sall4 genes. J Biol Chem. 2017;292:13045–13055. DOI: 10.1074/jbc.M117.777094 28623229 PMC5546042

[14] Kaya E, Doxzen KW, Knoll KR, et al. A bacterial Argonaute with noncanonical guide RNA specificity. Proc Natl Acad Sci U S A. 2016;113:4057–4062. DOI: 10.1073/pnas.1524385113 27035975 PMC4839417

[15] Qi J, Dong Z, Shi Y, et al. NgAgo-based fabp11a gene knockdown causes eye developmental defects in zebrafish. Cell Res. 2016;26:1349–1352. DOI: 10.1038/cr.2016.134 27834346 PMC5143420

[16] Staals RH, Zhu Y, Taylor DW, et al. RNA targeting by the type III-A CRISPR-Cas Csm complex of Thermus thermophilus. Mol Cell. 2014;56:518–530. DOI: 10.1016/j.molcel.2014.10.005 25457165 PMC4342149

[17] Tamulaitis G, Kazlauskiene M, Manakova E, et al. Programmable RNA shredding by the type III-A CRISPR-Cas system of *Streptococcus thermophilus*. Mol Cell. 2014;56:506–517. DOI: 10.1016/j.molcel.2014.09.027 25458845

[18] Hale CR, Zhao P, Olson S, et al. RNA-guided RNA cleavage by a CRISPR RNA-Cas protein complex. Cell. 2009;139:945–956. DOI: 10.1016/j.cell.2009.07.040 19945378 PMC2951265

[19] Zhang J, Rouillon C, Kerou M, et al. Structure and mechanism of the CMR complex for CRISPR-mediated antiviral immunity. Mol Cell. 2012;45:303–313. DOI: 10.1016/j.molcel.2011.12.013 22227115 PMC3381847

[20] Abudayyeh OO, Gootenberg JS, Konermann S, et al. C2c2 is a single-component programmable RNA-guided RNA-targeting CRISPR effector. Science. 2016;353:aaf5573 DOI: 10.1126/science.aaf5573 27256883 PMC5127784

[21] Shmakov S, Abudayyeh OO, Makarova KS, et al. Discovery and functional characterization of diverse class 2 CRISPR-Cas systems. Mol Cell. 2015;60:385–397. DOI: 10.1016/j.molcel.2015.10.008 26593719 PMC4660269

[22] East-Seletsky A, O'Connell MR, Knight SC, et al. Two distinct RNase activities of CRISPR-C2c2 enable guide-RNA processing and RNA detection. Nature. 2016;538:270–273. DOI: 10.1038/nature19802 27669025 PMC5576363

[23] Smargon AA, Cox DB, Pyzocha NK, et al. Cas13b Is a Type VI-B CRISPR-associated RNA-guided RNase differentially regulated by accessory proteins Csx27 and Csx28. Mol Cell. 2017;65:618–630 e617. DOI: 10.1016/j.molcel.2016.12.023 28065598 PMC5432119

[24] Yan WX, Chong S, Zhang H, et al. Cas13d is a compact RNA-targeting type VI CRISPR effector positively modulated by a WYL-domain-containing accessory protein. Mol Cell. 2018;70:327–339 e325. DOI: 10.1016/j.molcel.2018.02.028 29551514 PMC5935466

[25] O'Connell MR, Oakes BL, Sternberg SH, et al. Programmable RNA recognition and cleavage by CRISPR/Cas9. Nature. 2014;516:263–266. DOI: 10.1038/nature13769 25274302 PMC4268322

[26] Konermann S, Lotfy P, Brideau NJ, et al. Transcriptome engineering with RNA-targeting type VI-D CRISPR effectors. Cell. 2018;173:665–676 e614. DOI: 10.1016/j.cell.2018.02.033 29551272 PMC5910255

[27] Abudayyeh OO, Gootenberg JS, Essletzbichler P, et al. RNA targeting with CRISPR-Cas13. Nature. 2017;550:280–284. DOI: 10.1038/nature24049 28976959 PMC5706658

[28] Wolter F, Puchta H The CRISPR/Cas revolution reaches the RNA world: Cas13, a new Swiss Army knife for plant biologists. Plant J. 2018;94:767–775. DOI: 10.1111/tpj.13899 29575326

[29] Kushawah G, Abugattas-Nuñez del Prado J, Martinez-Morales JR, et al. CRISPR-Cas13d induces efficient mRNA knock-down in animal embryos. bioRxiv. 2020:2020.2001.2013.904763. DOI: 10.1101/2020.01.13.904763 32768421

[30] Rouillon C, Zhou M, Zhang J, et al. Structure of the CRISPR interference complex CSM reveals key similarities with cascade. Mol Cell. 2013;52:124–134. DOI: 10.1016/j.molcel.2013.08.020 24119402 PMC3807668

[31] Marraffini LA, Sontheimer EJ CRISPR interference limits horizontal gene transfer in staphylococci by targeting DNA. Science. 2008;322:1843–1845. DOI: 10.1126/science.1165771 19095942 PMC2695655

[32] Liu TY, Iavarone AT, Doudna JA RNA and DNA targeting by a reconstituted *Thermus thermophilus* type III-A CRISPR-Cas system. PLoS One. 2017;12:e0170552 DOI: 10.1371/journal.pone.0170552 28114398 PMC5256923

[33] Goldberg GW, Jiang W, Bikard D, et al. Conditional tolerance of temperate phages via transcription-dependent CRISPR-Cas targeting. Nature. 2014;514:633–637. DOI: 10.1038/nature13637 25174707 PMC4214910

[34] Kazlauskiene M, Tamulaitis G, Kostiuk G et al. Spatiotemporal control of type III-A CRISPR-Cas immunity: coupling DNA degradation with the target RNA recognition. Mol Cell. 2016;62:295–306. DOI: 10.1016/j.molcel.2016.03.024 27105119

[35] Samai P, Pyenson N, Jiang W, et al. Co-transcriptional DNA and RNA cleavage during type III CRISPR-Cas immunity. Cell. 2015;161:1164–1174. DOI: 10.1016/j.cell.2015.04.027 25959775 PMC4594840

[36] Aravind L, Koonin EV The HD domain defines a new superfamily of metal-dependent phosphohydrolases. Trends Biochem Sci. 1998;23:469–472. DOI: 10.1016/s0968-0004(98)01293-6 9868367

[37] Tamulaitis G, Venclovas C, Siksnys V Type III CRISPR-Cas immunity: major differences brushed aside. Trends Microbiol. 2017;25:49–61. DOI: 10.1016/j.tim.2016.09.012 27773522

[38] Hatoum-Aslan A, Maniv I, Samai P, et al. Genetic characterization of antiplasmid immunity through a type III-A CRISPR-Cas system. J Bacteriol. 2014;196:310–317. 10.1128/JB.01130-13 24187086 10.1128/JB.01130-13 PMC3911255

[39] Mogila I, Kazlauskiene M, Valinskyte S, et al. Genetic dissection of the type III-A CRISPR-Cas system csm complex reveals roles of individual subunits. Cell Rep. 2019;26:2753–2765 e2754. DOI: 10.1016/j.celrep.2019.02.029 30840895

[40] Krovel AV, Olsen LC Expression of a vas::EGFP transgene in primordial germ cells of the zebrafish. Mech Dev. 2002;116:141–150. DOI: 10.1016/s0925-4773(02)00154-5 12128213

[41] Kim MJ, Kang KH, Kim CH, et al. Real-time imaging of mitochondria in transgenic zebrafish expressing mitochondrially targeted GFP. Biotechniques. 2008;45:331–334. DOI: 10.2144/000112909 18778258

[42] Witzel HR, Jungblut B, Choe CP, et al. The LIM protein Ajuba restricts the second heart field progenitor pool by regulating Isl1 activity. Dev Cell. 2012;23:58–70. DOI: 10.1016/j.devcel.2012.06.005 22771034 PMC3671491

[43] Huang CJ, Tu CT, Hsiao CD, et al. Germ-line transmission of a myocardium-specific GFP transgene reveals critical regulatory elements in the cardiac myosin light chain 2 promoter of zebrafish. Dev Dyn. 2003;228:30–40. DOI: 10.1002/dvdy.10356 12950077

[44] Lawson ND, Weinstein BM In vivo imaging of embryonic vascular development using transgenic zebrafish. Dev Biol. 2002;248:307–318. DOI: 10.1006/dbio.2002.0711 12167406

[45] Zhang J, Talbot WS, Schier AF Positional cloning identifies zebrafish one-eyed pinhead as a permissive EGF-related ligand required during gastrulation. Cell. 1998;92:241–251. DOI: 10.1016/s0092-8674(00)80918-6 9458048

[46] Gritsman K, Zhang J, Cheng S, et al. The EGF-CFC protein one-eyed pinhead is essential for nodal signaling. Cell. 1999;97:121–132. DOI: 10.1016/s0092-8674(00)80720-5 10199408

[47] Griffin KJ, Kimelman D One-Eyed Pinhead and Spadetail are essential for heart and somite formation. Nat Cell Biol. 2002;4:821–825. DOI: 10.1038/ncb862 12360294

[48] Kazlauskiene M, Kostiuk G, Venclovas C, et al. A cyclic oligonucleotide signaling pathway in type III CRISPR-Cas systems. Science. 2017;357:605–609. DOI: 10.1126/science.aao0100 28663439

[49] Knaut H, Pelegri F, Bohmann K, et al. Zebrafish vasa RNA but not its protein is a component of the germ plasm and segregates asymmetrically before germline specification. J Cell Biol. 2000;149:875–888. DOI: 10.1083/jcb.149.4.875 10811828 PMC2174565

[50] Knaut H, Steinbeisser H, Schwarz H, et al. An evolutionary conserved region in the vasa 3'UTR targets RNA translation to the germ cells in the zebrafish. Curr Biol. 2002;12:454–466. DOI: 10.1016/s0960-9822(02)00723-6 11909530

[51] Braat AK, Zandbergen T, van de Water S, et al. Characterization of zebrafish primordial germ cells: morphology and early distribution of vasa RNA. Dev Dyn. 1999;216:153–167. DOI: 10.1002/(SICI)1097-0177(199910)216:2<153::AID-DVDY6>3.0.CO;2-1 10536055

[52] Huang N, Lee I, Marcotte EM, et al. Characterising and predicting haploinsufficiency in the human genome. PLoS Genet. 2010;6:e1001154 DOI: 10.1371/journal.pgen.1001154 20976243 PMC2954820

[53] Neuhauss SC, Biehlmaier O, Seeliger MW, et al. Genetic disorders of vision revealed by a behavioral screen of 400 essential loci in zebrafish. J Neurosci. 1999;19:8603–8615. DOI: 10.1523/JNEUROSCI.19-19-08603.1999 10493760 PMC6783047

[54] Gootenberg JS, Abudayyeh OO, Lee JW, et al. Nucleic acid detection with CRISPR-Cas13a/C2c2. Science. 2017;356:438–442. DOI: 10.1126/science.aam9321 28408723 PMC5526198

