# Supplementary material for: Targeted RNA Knockdown by a Type III CRISPR-Cas Complex in Zebrafish
Source: CRISPR J. 2020 Aug 24;3(4):299–313. doi: 10.1089/crispr.2020.0032 (PMC7469701; doi:10.1089/crispr.2020.0032)
Supplement: Supplemental data [file Supp_Data.pdf]

Supplementary methods for

Targeted RNA knockdown by crRNA guided Csm in zebrafish

Thomas Fricke[1,*], Dalia Smalakyte[2,*], Maciej Lapinski[1,*], Abhishek Pateria[1,*], Charles Weige[1], Michal Pastor[3], Agnieszka Kolano[1], Cecilia Winata[1], Virginijus Siksnys[2,$], Gintautas Tamulaitis[2,$], Matthias Bochtler[1,3,$]

[1]International Institute of Molecular and Cell Biology, Trojdena 4, 02-109 Warsaw, Poland
[2]Institute of Biotechnology, Vilnius University, Saulėtekio av. 7, 10257 Vilnius, Lithuania
[3]Polish Academy of Sciences, Institute of Biochemistry and Biophysics, Pawinskiego 5a, 02-106 Warsaw, Poland

EGFP and *avGFP* sequences

The target regions of the mRNAs of *avGFP* [1] and *EGFP* [2] used in this study span nucleotides 245-276 (**Figure 1B1**). In this region, the coding strands have the DNA sequences 5'-ACTTTTTCAAGAGTGCCATGCCCGAAGGTTAT-3' and 5'-ACTTCTTCAAGTCCGCCATGCCCGAAGGCTAC-3' differing in six positions (underlined) (also see **Table S2**).

tdgf1 sequences

To choose the most optimal crRNA sequences for the *tdgf1* transcript we considered the following features: specificity to the target sequence estimated with a BLAST (9254694) algorithm, avoidance of exon-exon boundaries, avoidance of annotated SNP sites, balanced GC content (>40%, <60%), low secondary structure probability as calculated by the RNAfold algorithm (with the -p1 parameter) from the Vienna RNA Package (22115189), low self-complementarity, and secondary structure probability of the crRNA as calculated by the RNAcofold algorithm.

In these regions, the coding strands have the DNA sequences 5'-ACGAAATGAACACGCAAACGCCGCAACGTCAA-3' (is targeted by crRNA *tdgf1*^{167}), 5'-GGTCAGAATGTGTGAAAGTTGGGGTTTCTGGA-3' (is targeted by crRNA *tdgf1*^{174}), 5'-AACAAAGCCGTACCTGCTGCAAGAATGGGGGA-3' (is targeted by crRNA *tdgf1*^{154}), 5'-TGATTATTTGTCAAGCTGTTTCACTCGAGTCA-3' (is targeted by crRNA *tdgf1*^{181}).

Artificial CRISPR loci

pCRISPR_*S3* plasmid containing four copies of *S3* spacer found in the CRISPR2 locus of *S. thermophilus* DGCC8004 was used for expression of *S3* crRNA [3]. For targeting *EGFP*, *avGFP* and *tdgf1* artificial CRISPR loci were constructed. A synthetic 445nt CRISPR loci containing four identical 36nt spacers of complementary sequence to the targeted region of *EGFP*, *avGFP* or *tdgf1*^{167} RNA separated by four 36nt repeats flanked by the leader sequence and the terminal repeat were cloned into the pACYC-Duet-1 vector to generate plasmids pCRISPR_*EGFP*, pCRISPR_*avGFP* and pCRISPR_ *tdgf1*^{167} (**Table S1**). For multiple targeting of *tdgf1*, a similar synthetic 733-nt CRISPR locus containing two identical 36nt spacers of complementary sequence to the targeted region of *tdgf1*^{181}, *tdgf1*^{174}, *tdgf1*^{167} and *tdgf1*^{154} RNAs separated by eight 36nt repeats flanked by the leader sequence and the terminal repeat was cloned into the pACYC-Duet-1 vector to generate a plasmid pCRISPR_*tdgf1*^{167,174,154,181}. The sequences of the leader and repeats were taken from CRISPR2 locus of *S. thermophilus* DGCC8004 [3]. Synthetic loci were obtained from GeneScript or Biocat. Full sequencing of cloned DNA fragments confirmed their identity to the original sequences.

Cloning, expression and purification of StCsm complexes

Wt and mutant StCsm complexes were obtained as described previously [3]. Synthetic CRISPR arrays were engineered to contain the 36 nt repeats naturally found in the CRISPR2 locus of *S. thermophilus* DGCC8004, separated by suitably chosen spacers. The resulting plasmids pCRISPR_*EGFP*, pCRISPR_*avGFP*, pCRISPR_*S3*, pCRISPR_*tdgf1*^{167} or pCRISPR_*tdgf1*^{181,174,167,154} were co-transformed with plasmid pCas/Csm (containing a cassette including all the *cas*/*csm* genes except *cas1* and *cas2*), and plasmid pCsm2-Tag (containing a N-terminal StrepII-tagged variant of the *csm2* gene) into *E. coli* ER2566 (DE3). Transformed cells were grown at 37°C in LB medium supplemented with streptomycin (25 µg/µl), ampicillin (50 µg/µl), and chloramphenicol (30 µg/µl) and expression of wt StCsm complex was induced using 1 mM IPTG. Further, StCsm complex was isolated by subsequent Strep-chelating affinity and size exclusion chromatography steps. The protein composition of the isolated StCsm was analyzed by SDS–PAGE Coomassie staining. StCsm complex containing dRNase Csm3 and dDNase Cas10 mutants were constructed and isolated as described earlier (17,28). *EGFP*-, *avGFP*-, *S3*-targeted StCsm complexes eluted from the columns were dialyzed against Tris Storage buffer (10 mM Tris-HCl (pH 8.5) buffer containing 300 mM KCl, 1 mM DTT, 0.1 mM EDTA, and 50% (v/v) glycerol) and stored at -20°C. StCsm complexes targeting *tdgf1* were eluted from the columns and dialyzed against PBS Storage buffer (1.06 mM KH_2PO4, 2.97mM Na_2HPO4 (pH 7.4) buffer containing 155 mM NaCl, 1 mM DTT and 50% (v/v) glycerol) and stored at -20°C. crRNAs co-purified with StCsm were isolated using phenol:chloroform:isoamylalcohol (25:24:1, v/v/v) extraction and precipitated with ethanol. crRNAs were separated on a denaturing 15% polyacrylamide gel (PAAG) and depicted with SybrGold (Thermo Scientific) staining.

Zebrafish lines and fish maintenance

Tg(ddx4:ddx4-EGFP) (ZFIN ID: ZDB-TGCONSTRCT-070814-1, germline) [2], *Tg(myl7:EGFP)* (ZFIN ID: ZDB-TGCONSTRCT-070117-164, heart) [4], and *Tg(nkx2.5:EGFP)* (ZFIN ID: ZDB-TGCONSTRCT-120828-5, heart) [5] fish were of ABTL genetic background. The *Tg(Xla.Eef1a1:mlsEGFP)* (ZFIN ID: ZDB-TGCONSTRCT-090309-1, throughout the embryo) [6] fish were of *nacre* genetic background [7]. The *Tg(fli1a:EGFP)* (ZFIN ID: ZDB-TGCONSTRCT-070117-94, blood vessels) [8] fish were of *casper* genetic background [9]. For *tdgf1* targeting, wild type AB fish maintained in the IIMCB Zebrafish Core Facility were used.

The nucleotide sequence of the *EGFP* transgenes was confirmed by Sanger sequencing in all cases. All reporter lines also contain wild-type copies of the respective genes. General maintenance, collection, and staging of the zebrafish were performed as described previously [10]. Embryos were maintained in E3 zebrafish medium at 28°C. The developmental stages were estimated based on time post-fertilization (hours or days; hpf or dpf) at 28°C.

Preparation of DNA and RNA substrates

Synthetic oligodeoxynucleotides were purchased from Metabion. All RNA substrates were obtained by *in vitro* transcription using TranscriptAid T7 High Yield Transcription Kit (Thermo Scientific). Briefly, plasmids pSG1154_avGFP, pUC18_S3/1 and pUC18_EGFP were used as a template to produce different DNA fragments by PCR using appropriate primers containing a T7 promoter in front of the desired RNA sequence. For the synthesis of *tdgf1* RNA substrates two annealed oligodeoxynucleotides containing T7 promoter and a target sequence were used as a template. RNA substrates were 5'-labeled with [γ^{32}P] ATP (Perkin Elmer) and PNK (Thermo Scientific). Ss M13mp18 plasmid DNA was purchased from New England BioLabs. A full description of RNA substrates is provided in Table S2.

Cleavage Assay *in vitro*

The StCsm RNA cleavage reactions *in vitro* were performed at 28°C and contained 8 nM of 5'-radiolabeled RNA and 160nM StCsm in the Reaction buffer (33 mM Tris-acetate (pH 7.9 at 25°C), 66 mM K-acetate, 0.1 mg/ml BSA) supplemented with 10mM Mg-acetate. Reactions were initiated by addition of the Mg^{2+}. The samples were collected at timed intervals and quenched by mixing 5 μl of reaction mixture with 10 μl of phenol:chloroform:isoamylalcohol (25:24:1, v/v/v). The aqueous phase was collected and mixed with 2x RNA loading buffer (Thermo Scientific) followed by incubation for 7 min at 85°C. The reaction products were separated on a denaturing 15% PAAG and depicted by autoradiography. The StCsm reactions on circular ssDNA *in vitro* were performed at 28°C and contained 1 nM M13mp18 ssDNA, 7.5 nM StCsm, and 7.5 nM RNA in the Reaction buffer supplemented with 10 mM $MnCl_2$. *tdgf1*-targeting StCsms present in PBS Storage buffer were firstly dyalized against Tris Reaction buffer (20 mM Tris-HCl (pH 8.5), 0.5 M NaCl, 1 mM EDTA, 7mM 2-mercaptoethanol) before DNA cleavage assay. Reactions were initiated by addition of Mn^{2+}. The samples were collected at timed intervals and quenched by mixing 5 μl of reaction mixture

with 2x loading dye (98% formaldehyde, 25 mM EDTA, 0.025% bromophenol blue), followed by incubation for 7 min at 85°C. The reaction products were separated during 1% agarose gel electrophoresis in TAE buffer (40 mM Tris, 5 mM Na-acetate, 0.9 mM EDTA, pH 7.9), stained with SYBR Gold (Thermo Scientific) and visualized using Fluorescent Image Analyzer FLA-2000 (Fuji Photo Film, Japan).

Microinjection

For *GFP* targeting, freshly laid fertilized eggs (or embryos) were collected from breeding tanks and injected with 1 nl of 0.5 mg/ml StCsm (if not stated otherwise) complex into the yolk of one cell stage embryos using an Eppendorf FemtoJet microinjection setup. For *tdgf1* targeting, the injection volume and the amount of StCsm complex had to be optimized. After injecting 1nl, 2nl, and 3nl of the StCsm ranging from 0.24 mg/ml to 1.9 mg/ml, the greatest penetrance was achieved by injecting a low volume (1 nl) of the highly concentrated (1.9 mg/ml) StCsm complex. Subsequent injections were only performed with injection volumes of 1 nl of StCsm complex, and the concentration of StCsm complexes was varied instead by diluting the stock with PBS storage buffer.

Microscopy

Embryos were anaesthetized using tricaine (MS-222). Fluorescence from live embryos was observed using a Leica M165FC fluorescent microscope equipped with a Leica DFC450C digital camera. After *tdgf1* targeting, embryos were anaesthetized using tricaine and removed carefully from the chorion using forceps under the microscope. Embryos were fixed in 2% methyl cellulose and imaged using a Leica M60 connected to a Leica DMC2900 camera. Images were acquired using Leica Application Suite v4.3 using standard settings.

Flow cytometry analysis

About 20-30 embryos at 48 hpf were collected randomly and washed twice with Hank's solution. The embryos were minced in a Petri dish with a fine scalpel. 800 µl of 0.25 % trypsin were added and mixed at 500 rpm on a bench top shaker for 1h at RT. The cells were filtered through a 40 µm cell strainer and washed 3 times with Hank's solution. The remaining was centrifuged at 2000 rpm. The pellet was resuspended in 500 µl Hank's solution and analyzed for EGFP fluorescence by BD FACS Calibur.

RNA-seq experiment and library preparation

For transcriptomic analysis, StCsm(*EGFP*) was diluted with water for injection(1:1 v/v) up to a concentration of 0.7mg/ml. For mock injection, Tris storage buffer was also diluted in the similar manner and 1nl (0.7ng) of either StCsm(*EGFP*) or buffer alone were injected into the yolk of embryos at the 1-cell stage. For each group, three replicates of 25 pooled embryos each were isolated and their total RNA extracted at three different time points: 128-cell, 5 hpf, and 24 hpf (pair injection and mock injection samples were always randomly chosen from pooled offspring from three different mating pairs). RNA extraction was performed

using Tri Reagent (Invitrogen) and purified with Zymo RNA Clean and Concentrator-5 kit according to the manufacturers' protocol. Subsequently, 1.5 µg of total RNA was subjected to Lexogen poly(A) RNA selection kit and the poly(A)-selected mRNAs were used for RNA-seq library construction using the CORALL Total RNA-seq Library Preparation Kit (Lexogen) according to the manufacturer's protocol. Sequencing was performed on a Nextseq 500 on a high output kit with paired-end reads. Read 1 had 71 bases in length, while read 2 had 59.

RNA sequencing data pre-processing

Base-calling and demultiplexing were performed with Illumina's bcl2fastq v2.16.0.10. The data processing pipeline was written in Nextflow [11]. The code is available on GitLab: https://gitlab.com/lapinskim/csm_sequencing_analysis. The quality of sequencing reads, and alignments were assessed with FastQC v. 0.11.8 and MultiQC v. 1.7 [12]. Low-quality sequences and adapter content were trimmed with cutadapt v. 2.4 (10.14806/ej.17.1.200) following the recommendations of the manufacturer of the total RNA sample prep kit (https://www.lexogen.com/wp-content/uploads/2019/02/095UG190V0110_CORALL-Total-RNA-Seq.pdf#page=34). Reads were aligned to the GRCz11 reference genome supplemented with the *EGFP* sequence with STAR v. 2.7.2b [13] using the following custom parameters: --outFilterType BySJout --outFilterMultimapNmax 20 --alignSJoverhangMin 8 --alignSJDBoverhangMin 1 --outFilterMismatchNmax 999 --alignIntronMin 20 --alignIntronMax 1000000 --alignMatesGapMax 1000000 --outFilterMismatchNoverLmax 0.6. Alignments sharing the same position were de-duplicated based on their Unique Molecular Identifier sequence using UMI-tools v. 1.0.0 [14]. We counted the de-duplicated alignments per exon on the gene-level with the htseq-count script from the HTSeq v. 0.11.2 python package [15] utilizing the Ensembl GRCz11 version 97 genome annotation. .

RNA sequencing data analysis

The pre-processed sequencing data was analyzed in the R v. 3.6.1 programming language [16]. The script containing the analysis is available on GitLab: https://gitlab.com/lapinskim/csm_data_analysis. Differential expression analysis was performed using DESeq2 package [17] with apeglm [18] for the log fold change. Principal component analysis was performed on the normalized read counts, transformed with the variance stabilizing transformation from the same package.

We checked if the Csm injection had an effect on the *EGFP* expression, represented by the number of sequencing reads aligned to the *EGFP* transcript. We fitted two models to our dataset: a reduced model, stating that the log2 transformed counts are dependent on the batch effect only, as well as a full model stating that the log2 transformed count are dependent on experimental condition in addition to the batch effect. We compared the goodness of fit of the two models using the likelihood ratio test.

For *EGFP* coverage analysis, we loaded the alignments with the GenomicAlignments [19] and visualized them with Gviz [20] package. The coverage at each base position of the *EGFP* transcript was calculated with the IRanges package [19]. For plotting, the coverage values of each replica sample were scaled by maximum, to be in the range of 0 to 1. To visualize the coverage differences, we calculated the log2 transformed ratio of scaled coverage values of Csm injected samples to those injected with mock. Finally, a mean fraction of total sequencing fragment ends was calculated at each base position of the *EGFP* transcript for all experimental conditions. To visualize the differences in sequencing fragment end distribution, the calculated values for the mock condition were subtracted from those coming from the Csm condition.

The enrichment of biological process Gene Ontology terms, within the differentially expressed gene set at 128-cell stage, was calculated using ClusterProfiler R package [21]. The *enrichGO* function was utilized with the parameters *pvalueCutoff* equal to 0.01 and *qvalueCutoff* equal to 0.05.

To assess whether the genes from the differentially expressed group at 128-cell stage contain sequence homologous to the crRNA we searched zebrafish transcriptome with the crRNA sequence using the BLAST algorithm version 2.9.0 [22]. To find the best high-scoring pair for each transcript, the following parameters were used: -evalue 1000, -word_size 4, -subject_besthit, -max_hsps 1, max_target_seqs 10000. The pair with the highest number of identical nucleotides for each gene were considered in further analysis. We then hypothesized that, if these transcripts were targeted by the crRNA, their decrease should correlate significantly with the number of identical nucleotides. To test this, we performed an analysis of variance to check if the model with the number of identical nucleotides between the transcript and crRNA explains the log2 fold change in the number of aligned reads better than the one without it. Moreover, to enhance the statistical power, we performed the analysis on genes cumulatively with every decrease in identical nucleotide numbers. We performed analysis of variance on the group with maximum homology (22 nucleotides), each time expanding it, by lowering the number of identical nucleotides required to qualify for the group by one, to see if this improves the test statistic.

References

1. Chalfie M, Tu Y, Euskirchen G et al. Green fluorescent protein as a marker for gene expression. *Science.* 1994;263:802-805. DOI: 10.1126/science.8303295
2. Krovel AV, Olsen LC. Expression of a vas::EGFP transgene in primordial germ cells of the zebrafish. *Mech Dev.* 2002;116:141-150.
3. Tamulaitis G, Kazlauskiene M, Manakova E et al. Programmable RNA shredding by the type III-A CRISPR-Cas system of Streptococcus thermophilus. *Mol Cell.* 2014;56:506-517. DOI: 10.1016/j.molcel.2014.09.027
4. Huang CJ, Tu CT, Hsiao CD et al. Germ-line transmission of a myocardium-specific GFP transgene reveals critical regulatory elements in the cardiac myosin light chain 2 promoter of zebrafish. *Dev Dyn.*

2003;228:30-40. DOI: 10.1002/dvdy.10356
5. Witzel HR, Jungblut B, Choe CP et al. The LIM protein Ajuba restricts the second heart field progenitor pool by regulating Isl1 activity. *Dev Cell.* 2012;23:58-70. DOI: 10.1016/j.devcel.2012.06.005
6. Kim MJ, Kang KH, Kim CH et al. Real-time imaging of mitochondria in transgenic zebrafish expressing mitochondrially targeted GFP. *Biotechniques.* 2008;45:331-334. DOI: 10.2144/000112909
7. Lister JA, Robertson CP, Lepage T et al. nacre encodes a zebrafish microphthalmia-related protein that regulates neural-crest-derived pigment cell fate. *Development.* 1999;126:3757-3767.
8. Lawson ND, Weinstein BM. In vivo imaging of embryonic vascular development using transgenic zebrafish. *Dev Biol.* 2002;248:307-318.
9. White RM, Sessa A, Burke C et al. Transparent adult zebrafish as a tool for in vivo transplantation analysis. *Cell Stem Cell.* 2008;2:183-189. DOI: 10.1016/j.stem.2007.11.002
10. Westerfield M. The zebrafish book : a guide for the laboratory use of zebrafish (Brachydanio rerio). Eugene, OR: M. Westerfield. 1993.
11. Di Tommaso P, Chatzou M, Floden EW et al. Nextflow enables reproducible computational workflows. *Nat Biotechnol.* 2017;35:316-319. DOI: 10.1038/nbt.3820
12. Ewels P, Magnusson M, Lundin S et al. MultiQC: summarize analysis results for multiple tools and samples in a single report. *Bioinformatics.* 2016;32:3047-3048. DOI: 10.1093/bioinformatics/btw354
13. Dobin A, Davis CA, Schlesinger F et al. STAR: ultrafast universal RNA-seq aligner. *Bioinformatics.* 2013;29:15-21. DOI: 10.1093/bioinformatics/bts635
14. Smith T, Heger A, Sudbery I. UMI-tools: modeling sequencing errors in Unique Molecular Identifiers to improve quantification accuracy. *Genome Res.* 2017;27:491-499. DOI: 10.1101/gr.209601.116
15. Anders S, Pyl PT, Huber W. HTSeq--a Python framework to work with high-throughput sequencing data. *Bioinformatics.* 2015;31:166-169. DOI: 10.1093/bioinformatics/btu638
16. Team RC. A Language and Environment for Statistical Computing. https://wwwr-projectorg. 2019.
17. Love MI, Huber W, Anders S. Moderated estimation of fold change and dispersion for RNA-seq data with DESeq2. *Genome Biol.* 2014;15:550. DOI: 10.1186/s13059-014-0550-8
18. Zhu A, Ibrahim JG, Love MI. Heavy-tailed prior distributions for sequence count data: removing the noise and preserving large differences. *Bioinformatics.* 2019;35:2084-2092. DOI: 10.1093/bioinformatics/bty895
19. Lawrence M, Huber W, Pages H et al. Software for computing and annotating genomic ranges. *PLoS Comput Biol.* 2013;9:e1003118. DOI: 10.1371/journal.pcbi.1003118
20. Hahne F, Ivanek R. Visualizing Genomic Data Using Gviz and Bioconductor. *Methods Mol Biol.* 2016;1418:335-351. DOI: 10.1007/978-1-4939-3578-9_16
21. Yu G, Wang LG, Han Y et al. clusterProfiler: an R package for comparing biological themes among gene clusters. *OMICS.* 2012;16:284-287. DOI: 10.1089/omi.2011.0118
22. Altschul SF, Madden TL, Schaffer AA et al. Gapped BLAST and PSI-BLAST: a new generation of protein database search programs. *Nucleic Acids Res.* 1997;25:3389-3402. DOI: 10.1093/nar/25.17.3389
