# Supplementary material for: Targeted RNA Knockdown by a Type III CRISPR-Cas Complex in Zebrafish
Source: CRISPR J. 2020 Aug 24;3(4):299–313. doi: 10.1089/crispr.2020.0032 (PMC7469701; doi:10.1089/crispr.2020.0032)
Supplement: Supplemental data [file Supp_Table1.docx]

**Table S1. Expression plasmids used in this study.** **Related to Figures 1 and 4.**

| **Plasmid** | **Description*** | **Usage** |
| --- | --- | --- |
| pCas/Csm | pCDFDuet-1_Cas6-Cas10-Csm2-Csm3-Csm4-Csm5-Csm6’-Csm6 (Str^r^) | expression of wt *S. thermophilus* DGCC8004 StCsm complexes |
| pCsm2N-Tag | pETDuet-1_Csm2-N-StrepII (Ap^r^) | expressed Csm2 N-StrepII-tagged protein was used as a bait for StCsm pulldown |
| pCRISPR_*EGFP* | pACYCDuet-1_Leader-(repeat_spacer *EGFP*)_4_-terminal repeat (Cm^r^) | expression of crRNA containing *S. thermophilus* DGCC8004 repeat and 36 nt length spacer targeting *EGFP* transcript |
| pCRISPR_*avGFP* | pACYCDuet-1_Leader-(repeat_spacer *avGFP*)_4_-terminal repeat (Cm^r^) | expression of crRNA containing *S. thermophilus* DGCC8004 repeat and 36 nt length spacer targeting *avGFP* transcript |
| pCRISPR_*S3* | pACYCDuet-1_Leader-(repeat_spacer *S3*)_4_-terminal repeat (Cm^r^) | expression of *S. thermophilus* DGCC8004 crRNA containing 36 nt length spacer *S3* sequence |
| pCRISPR_*tdgf1^167^* | pACYCDuet-1_Leader-(repeat_spacer *tdgf1^167^*)_4_-terminal repeat (Cm^r^) | expression of crRNA containing *S. thermophilus* DGCC8004 repeat and 36nt length spacer targeting *tdgf1* transcript at position 149-180 nt and *S. thermophilus* DGCC8004 repeat |
| pCRISPR_*tdgf1^167,174,154,181^* | pACYCDuet-1_Leader-(repeat_spacer *tdgf1^167^*_repeat_spacer *tdgf1^174^*_repeat_spacer *tdgf1^154^*_repeat_spacer *tdgf1^181^*)_2_-terminal repeat (Cm^r^) | expression of crRNAs containing *S. thermophilus* DGCC8004 repeats and 36nt length spacers targeting *tdgf1* transcript at positions 149-180 nt, 80-111 nt, 233-264nt and 18-49 nt, respectively |

* Str^r^ – streptomycin-resistance, Ap^r^ – ampicillin-resistance, Cmr^r^ – chloramphenicol-resistance.
