# Supplementary material for: Targeted RNA Knockdown by a Type III CRISPR-Cas Complex in Zebrafish
Source: CRISPR J. 2020 Aug 24;3(4):299–313. doi: 10.1089/crispr.2020.0032 (PMC7469701; doi:10.1089/crispr.2020.0032)
Supplement: Supplemental data [file Supp_Table2.docx]

**Table S2. crRNAs targeting *EGFP, avGFP*, *S3* and *tdgf1* and RNA substrates used in this study*. Related to Figures 1, 3 and 4.**

* Above each Table *EGFP, avGFP*, *S3* and *tdgf1* targeting crRNAs are depicted. Bold and underlined lettering in crRNAs indicates the spacer and 5’- handle sequences, respectively. Target sequences in *EGFP, avGFP* and *S3* RNA substrates containing the corresponding transcript sequences of are coloured in green, olive and red, respectively. Different target sequences in *tdgf1* RNA substrates are coloured in violet, yellow, brown and blue. Nucleotides complementary to corresponding nucleotide in crRNA are depicted by dashes. “:” indicates nucleotides which form wobble base pair. First 5‘-GGG nucleotides were incorporated into RNA substrates during *in vitro* transcription. Most stable hetero-dimers are shown for NS RNA and *tdgf1* RNAs, *S3* RNA and *EGFP* RNA.

| **72 nt *EGFP* crRNA in StCsm complex** | | 5'-handle Spacer(*EGFP*) 3'-handle  5’-ACGGAAAC**GUAGCCUUCGGGCAUGGCGGACUUGAAGAAGUCGUG**GAUAUAAACCUAAUUACCUCGAGAGGGG-3’ |
| --- | --- | --- |
| **40 nt *EGFP* crRNA in StCsm complex** | | 5'-handle Spacer(*EGFP*)  5’-ACGGAAAC**GUAGCCUUCGGGCAUGGCGGACUUGAAGAAGU**-3’ |
| **Substrate** | **Length, nt** | **Sequence** |
| *EGFP* RNA | 70 | \| \|\|\|\|\|\|\|\|\|\|\|\|\|\|\|\|\|\|\|\|\|\|\|\|\|\|\|\|\|\|\|\|\|\|  3’-GCGAGGACCUG**CAUCGGAAGCCCGUACCGCCUGAACUUCUUCA**GCACGACGAAGUACACCAGCCCCAGGG-5’ |
|  | | |
| **72 nt *avGFP* crRNA in StCsm complex** | | 5'-handle Spacer(*avGFP*) 3'-handle  5’- ACGGAAAC**AUAACCUUCGGGCAUGGCACUCUUGAAAAAGUCAUG**GAUAUAAACCUAAUUACCUCGAGAGGGG-3’ |
| **40 nt *avGFP* crRNA in StCsm complex** | | 5'-handle Spacer(*avGFP*)  5’- ACGGAAAC**AUAACCUUCGGGCAUGGCACUCUUGAAAAAGU**-3’ |
| **Substrate** | **Length, nt** | **Sequence** |
| *avGFP* RNA | 68 | \| \|\|\|\|\|\|\|\|\|\|\|\|\|\|\|\|\|\|\|\|\|\|\|\|\|\|\|\|\|\|\|\|\|\|  3’-UCAAGAAAGGACAUG**UAUUGGAAGCCCGUACCGUGAGAACUUUUUCA**GUACGACAAAGUAUACUAGGG-5’ |
| *EGFP* RNA | 70 | \| \|\| \|\| \|\|\|\|\|\|\|\|\|\|\|\|\|\| \|\|\|\|\|\| \|\|\|\|  3’-GCGAGGACCUG**CAUCGGAAGCCCGUACCGCCUGAACUUCUUCA**GCACGACGAAGUACACCAGCCCCAGGG-5’ |

| **72 nt *S3* crRNA in StCsm complex** | | 5'-handle Spacer(*S3)* 3'-handle  5’-ACGGAAAC**UUUCGUAACUGUUUAAUUCUGUUCACUUAUUCCACC**GAUAUAAACCUAAUUACCUCGAGAGGGG-3’ |
| --- | --- | --- |
| **40 nt *S3* crRNA in StCsm complex** | | 5'-handle Spacer(*S3*)  5'-ACGGAAAC**UUUCGUAACUGUUUAAUUCUGUUCACUUAUUC**-3' |
| **Substrate** | **Length, nt** | **Sequence** |
| *S3* RNA | 68 | \|\|\|\|\|\|\|\|\|\|\|\|\|\|\|\|\|\|\|\|\|\|\|\|\|\|\|\|\|\|\|\|  3’-AGCGGUGGAAAACAA**AAAGCAUUGACAAAUUAAGACAAGUGAAUAAG**GUGGAGGAGUUAAACGGCGGG-5’ |
| *EGFP* RNA | 70 | \| \|\| \| : \| \| : : ::\| \|  3’-GCGAGGACCUG**CAUCGGAAGCCCGUACCGCCUGAACUUCUUCA**GCACGACGAAGUACACCAGCCCCAGGG-5’ |

| **72 nt *tdgf1^167^* crRNA in StCsm complex** | | 5'-handle Spacer(*tdgf1^167^*)  5'-ACGGAAAC**UUGACGUUGCGGCGUUUGCGUGUUCAUUUCGUUAAA**GAUAUAAACCUAAUUACCUCGAGAGGGG-3' |
| --- | --- | --- |
| **40 nt *tdgf1^167^* crRNA in StCsm complex** | | 5'-handle Spacer(*tdgf1^167^*)  5'-ACGGAAAC**UUGACGUUGCGGCGUUUGCGUGUUCAUUUCGU**-3' |
| **Substrate** | **Length, nt** | **Sequence** |
| *tdgf1^167^* RNA | 43 | \| \|\|\|\|\|\|\|\|\|\|\|\|\|\|\|\|\|\|\|\|\|\|\|\|\|\|\|\|\|\|\|\|  3’-AACGCCAC**AACUGCAACGCCGCAAACGCACAAGUAAAGCA**GGG-5’ |
| NS RNA | 68 | :: \| \| : \| \| :\|\|\|\|\| ::  3’-AGCGGUGGAAAACAACCACCUUAUUCACUUGUCUUAAUUUGUCAAUGCUUUAGGAGUUAAACGGCGGG-5’ |
|  | |  |
| **72 nt *tdgf1^174^* crRNA in StCsm complex** | | 5'-handle Spacer(*tdgf1^174^*)  5'-ACGGAAAC**UCCAGAAACCCCAACUUUCACACAUUCUGACCCCUC**GAUAUAAACCUAAUUACCUCGAGAGGGG-3' |
| **40 nt *tdgf1*^174^ crRNA in StCsm complex** | | 5'-handle Spacer(*tdgf1^174^*)  5'-ACGGAAAC**UCCAGAAACCCCAACUUUCACACAUUCUGACC**-3' |
| **Substrate** | **Length, nt** | **Sequence** |
| *tdgf1^174^* RNA | 43 | \| \|\|\|\|\|\|\|\|\|\|\|\|\|\|\|\|\|\|\|\|\|\|\|\|\|\|\|\|\|\|\|\|  3’-AAACCGAA**AGGUCUUUGGGGUUGAAAGUGUGUAAGACUGG**GGG-5’ |
| NS RNA | 68 | \| :\| \|\|\|\|\| \| \|\| \| \| :: ::\|  3’-AGCGGUGGAAAACAACCACCUUAUUCACUUGUCUUAAUUUGUCAAUGCUUUAGGAGUUAAACGGCGGG-5’ |
|  | |  |
| **72 nt *tdgf1^154^* crRNA in StCsm complex** | | 5'-handle Spacer(*tdgf1^154^*)  5'-ACGGAAAC**UCCCCCAUUCUUGCAGCAGGUACGGCUUUGUUUGGC**GAUAUAAACCUAAUUACCUCGAGAGGGG-3' |
| **40 nt *tdgf1^154^* crRNA in StCsm complex** | | 5'-handle Spacer(*tdgf1^154^*)  5'-ACGGAAAC**UCCCCCAUUCUUGCAGCAGGUACGGCUUUGUU**-3' |
| **Substrate** | **Length, nt** | **Sequence** |
| *tdgf1^154^* RNA | 43 | \| \| \| \|\|\|\|\|\|\|\|\|\|\|\|\|\|\|\|\|\|\|\|\|\|\|\|\|\|\|\|\|\|\|\|  3’-UACGUGCA**AGGGGGUAAGAACGUCGUCCAUGCCGAAACAA**GGG-5’ |
| NS RNA | 68 | \|\| \|\|\|\|\|\|  3’-AGCGGUGGAAAACAACCACCUUAUUCACUUGUCUUAAUUUGUCAAUGCUUUAGGAGUUAAACGGCGGG-5’ |
|  | |  |
| **72 nt *tdgf1^181^* crRNA in StCsm** | | 5'-handle Spacer (*tdgf1^181^*)  5'-ACGGAAAC**UGACUCGAGUGAAACAGCUUGACAAAUAAUCACAGC**GAUAUAAACCUAAUUACCUCGAGAGGGG-3' |
| **40 nt *tdgf1^181^* crRNA in StCsm** | | 5'-handle Spacer (*tdgf1^181^*)  5'-ACGGAAAC**UGACUCGAGUGAAACAGCUUGACAAAUAAUCA**-3' |
| **Substrate** | **Length, nt** | **Sequence** |
| *tdgf1^181^* RNA | 43 | \|: \| \|\|\|\|\|\|\|\|\|\|\|\|\|\|\|\|\|\|\|\|\|\|\|\|\|\|\|\|\|\|\|\|\|  3’-AGUGUAGG**ACUGAGCUCACUUUGUCGAACUGUUUAUUAGU**GGG-5’ |
| NS RNA | 68 | :: \|\| \|\|\| : \|\|:\| \|\|\|\|\|\| \| \|\| : : \|  3’-AGCGGUGGAAAACAACCACCUUAUUCACUUGUCUUAAUUUGUCAAUGCUUUAGGAGUUAAACGGCGGG-5’ |
