# Supplementary material for: Targeted RNA Knockdown by a Type III CRISPR-Cas Complex in Zebrafish
Source: CRISPR J. 2020 Aug 24;3(4):299–313. doi: 10.1089/crispr.2020.0032 (PMC7469701; doi:10.1089/crispr.2020.0032)

Supplemental Figures

Figure S1: *In vitro* characterization of StCsm complexes targeting *EGFP* transcript. Related to Figure 1. (A) Coomassie blue-stained SDS-PAGE of purified StCsm complexes. M - protein mass marker. (B) Denaturing PAGE analysis of crRNA co-purifying with the StCsm complexes. StCsm complex co-purifies with two different RNAs: unmatured 72 nt cRNA or matured 40 nt crRNA. 72 nt crRNA resulting from the pre-crRNA cleavage is comprised of 8 nt 5'-handle, 36 nt spacer and 28 nt 3'-handle. Matured 40 nt crRNA contains an 8 nt 5'-handle derived from the repeat sequence and a 32 nt spacer which resulted from the 3'-end processing of unmatured 72 nt crRNA.

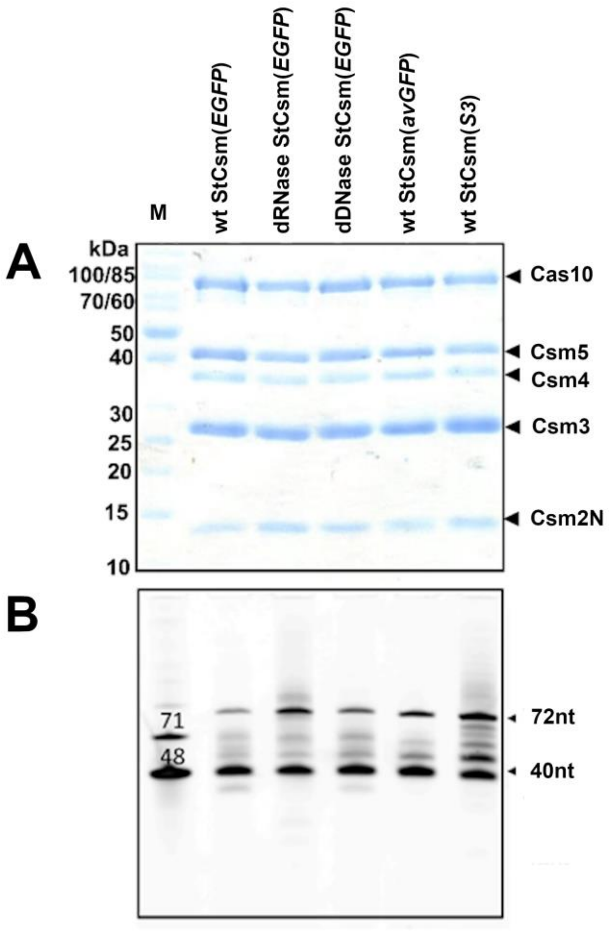

Supplement: Supplemental data [file Supp_Fig1.pdf]
