# Supplementary material for: Targeted RNA Knockdown by a Type III CRISPR-Cas Complex in Zebrafish
Source: CRISPR J. 2020 Aug 24;3(4):299–313. doi: 10.1089/crispr.2020.0032 (PMC7469701; doi:10.1089/crispr.2020.0032)

Figure S2: In vitro RNase and DNase assays. Related to Figure 1. (A) Cartoon of the RNA cleavage assay. (B) Results of the RNA cleavage assay. *In vitro* synthesized radioactively 5'-labeled segment of *EGFP* transcript was used as a substrate in RNA cleavage assay. Reactions were initiated by addition of Mg^{2+} and products were analyzed in denaturing polyacrylamide gel. (C) Cartoon of the DNA cleavage assay. Binary StCsm complex binds complementary target RNA and forms ternary StCsm complex. The binding of complementary target RNA activates Cas10 (pink circle) and ssDNA is degraded. (D) Results of the DNA cleavage assay. Reactions were initiated by addition of Mn^{2+} and products were analyzed on agarose gels.

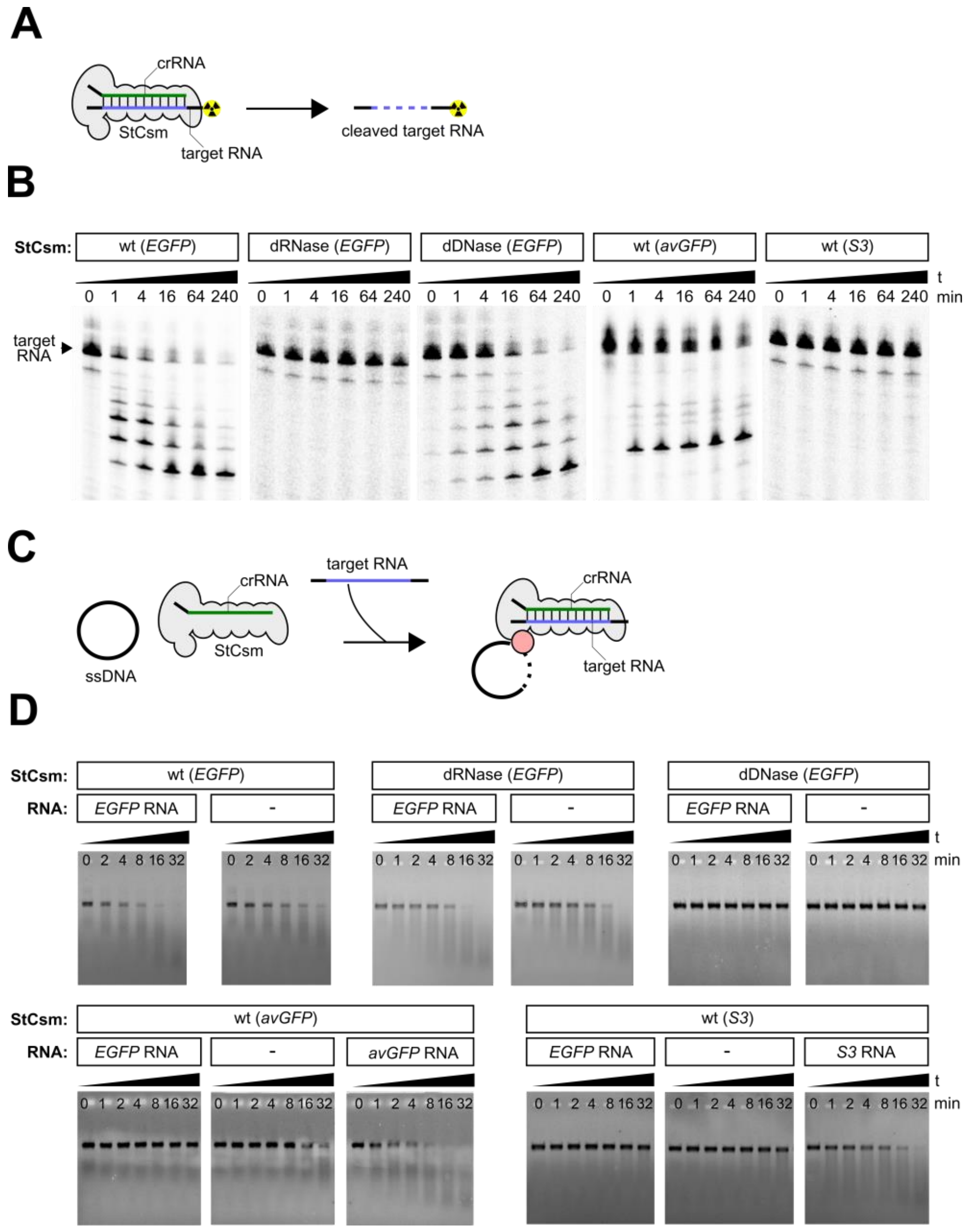

Supplement: Supplemental data [file Supp_Fig2.pdf]
