# Supplementary material for: Targeted RNA Knockdown by a Type III CRISPR-Cas Complex in Zebrafish
Source: CRISPR J. 2020 Aug 24;3(4):299–313. doi: 10.1089/crispr.2020.0032 (PMC7469701; doi:10.1089/crispr.2020.0032)

Figure S3: *In vitro* characterization of StCsm complexes targeting *tdgf1* transcript. Related to Figure 4. (A) Coomassie blue-stained SDS-PAGE of purified StCsm complexes. M - protein mass marker. (B) Denaturing PAGE analysis of crRNA co-purifying with the StCsm complexes. 0 – crRNA from wt StCsm(*S3*) used as marker. (C) RNA cleavage activity *in vitro* by single and multiple *tdgf1*-targeting StCsm complexes. Depletion of *in vitro* transcribed radioactively 5'-labeled target RNAs measured in 45 minutes after RNA cleavage reaction initiation with Mg^{2+}. Cleavage reactions were performed at 28°C and contained 8 nM of 5'-radiolabeled RNA, 160 nM StCsm and 10mM Mg-acetate. For wt-StCsm(*tdgf1*^{167}) only *tdgf1*^{167} RNA and NS RNA substrates were used (**Table S2**). Data are represented as mean ± SEM. (D) DNA cleavage *in vitro* by wt, dRNase, and dDNase StCsm(*tdgf1*^{167,174,154,181}) complexes in the presence of the *tdgf1*^{167} RNA. Cleavage reactions were performed at 28°C and contained 1 nM M13mp18 ssDNA, 7.5 nM StCsm, 10 mM MnCl$_2$ and 7.5 nM *tdgf1*^{167} RNA. Reactions were initiated by addition of Mn^{2+}. Products were analyzed on agarose gel.

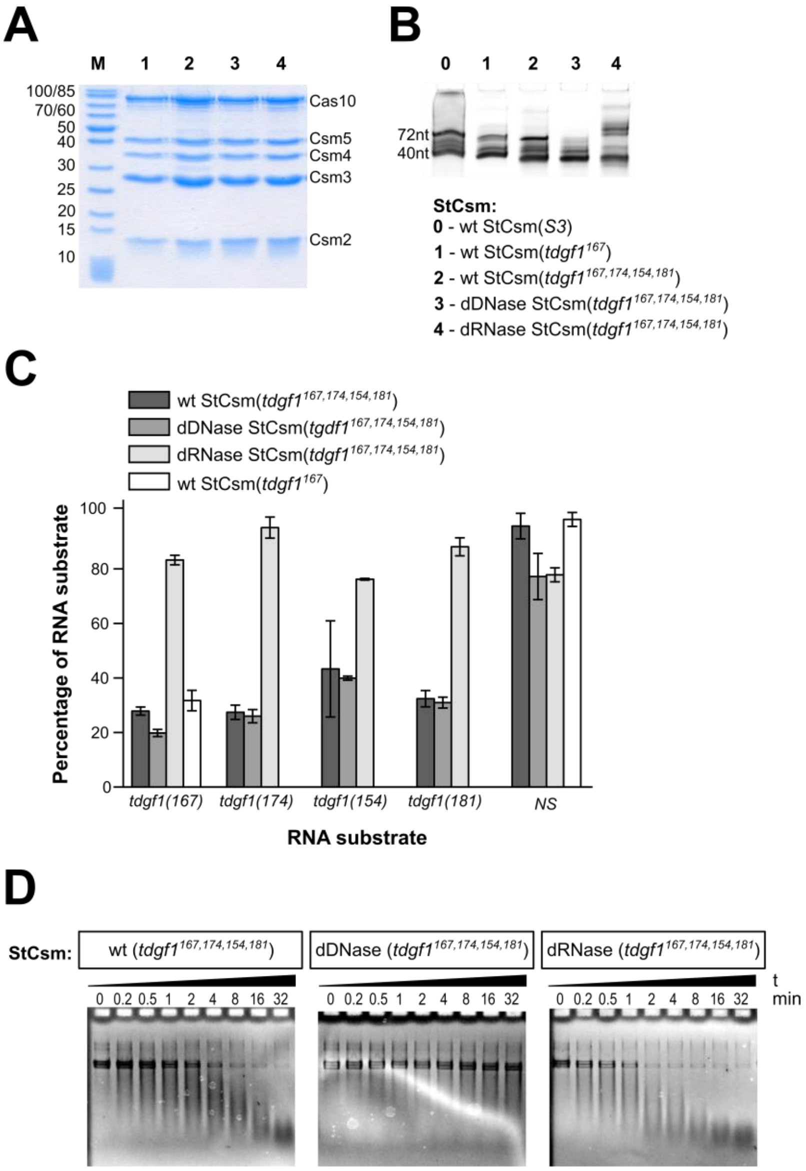

Supplement: Supplemental data [file Supp_Fig3.pdf]
