# Supplementary material for: Targeted RNA Knockdown by a Type III CRISPR-Cas Complex in Zebrafish
Source: CRISPR J. 2020 Aug 24;3(4):299–313. doi: 10.1089/crispr.2020.0032 (PMC7469701; doi:10.1089/crispr.2020.0032)

**Figure S4: *Tg(ddx4:ddx4-EGFP)* in reciprocal crosses. Related to Figure 3.** *Tg(ddx4:ddx4-EGFP)* fluorescence was monitored in reciprocal crosses of ABTL and *Tg(ddx4:ddx4-EGFP)* parents.

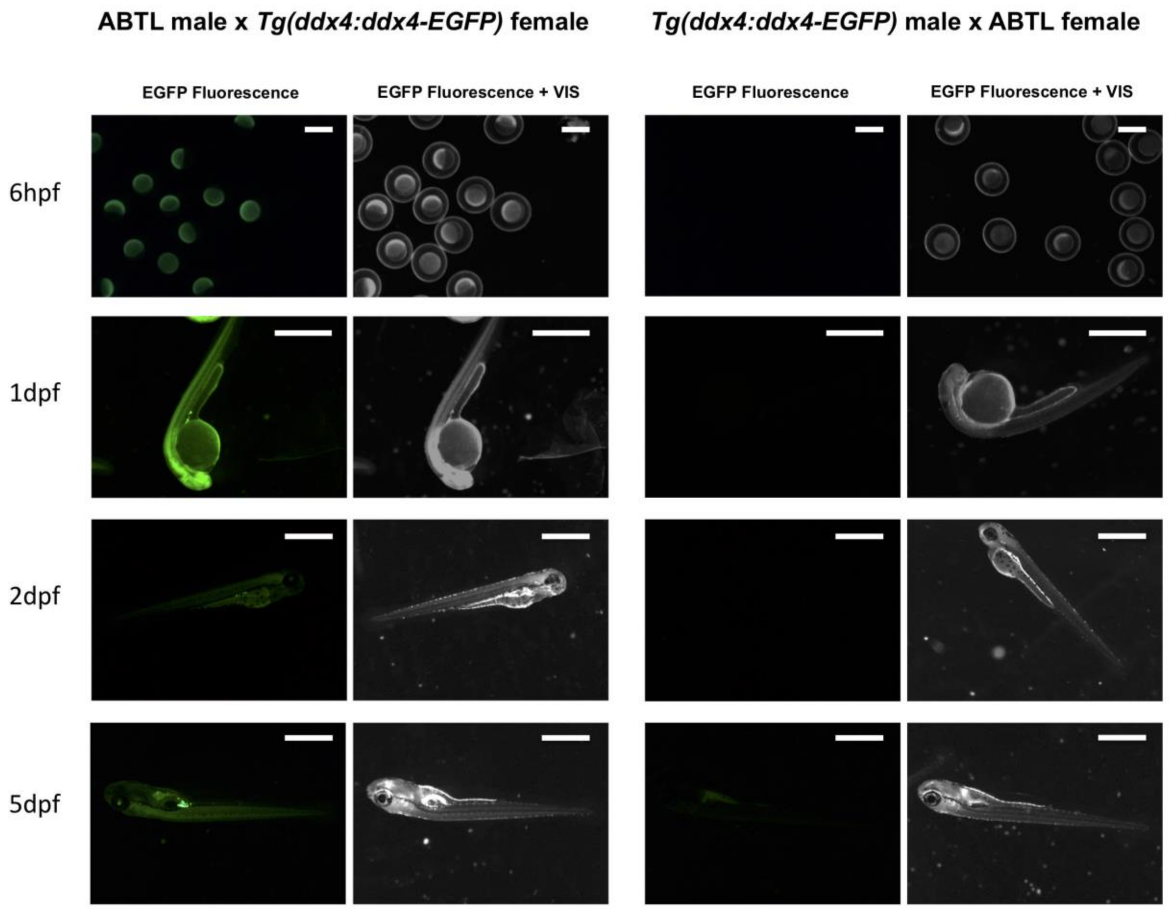

Supplement: Supplemental data [file Supp_Fig4.pdf]
