# Supplementary material for: Targeted RNA Knockdown by a Type III CRISPR-Cas Complex in Zebrafish
Source: CRISPR J. 2020 Aug 24;3(4):299–313. doi: 10.1089/crispr.2020.0032 (PMC7469701; doi:10.1089/crispr.2020.0032)

Figure S5: Lack of toxicity and dose response. Related to Figure 3. (A) StCsm(*EGFP*) complexes were tolerated up to the highest tested does of 2.5 ng, or 5-times the efficacious does. Even at the high dose, no overt phenotypic changes were observed. Arrows point to germ cells. (B) The dose dependence of *Tg(ddx4:ddx4-EGFP)* knockdown was monitored for 1nl injections. At 0.5 mg/ml, the majority of embryos did not show EGFP fluorescence in germ cells.

-

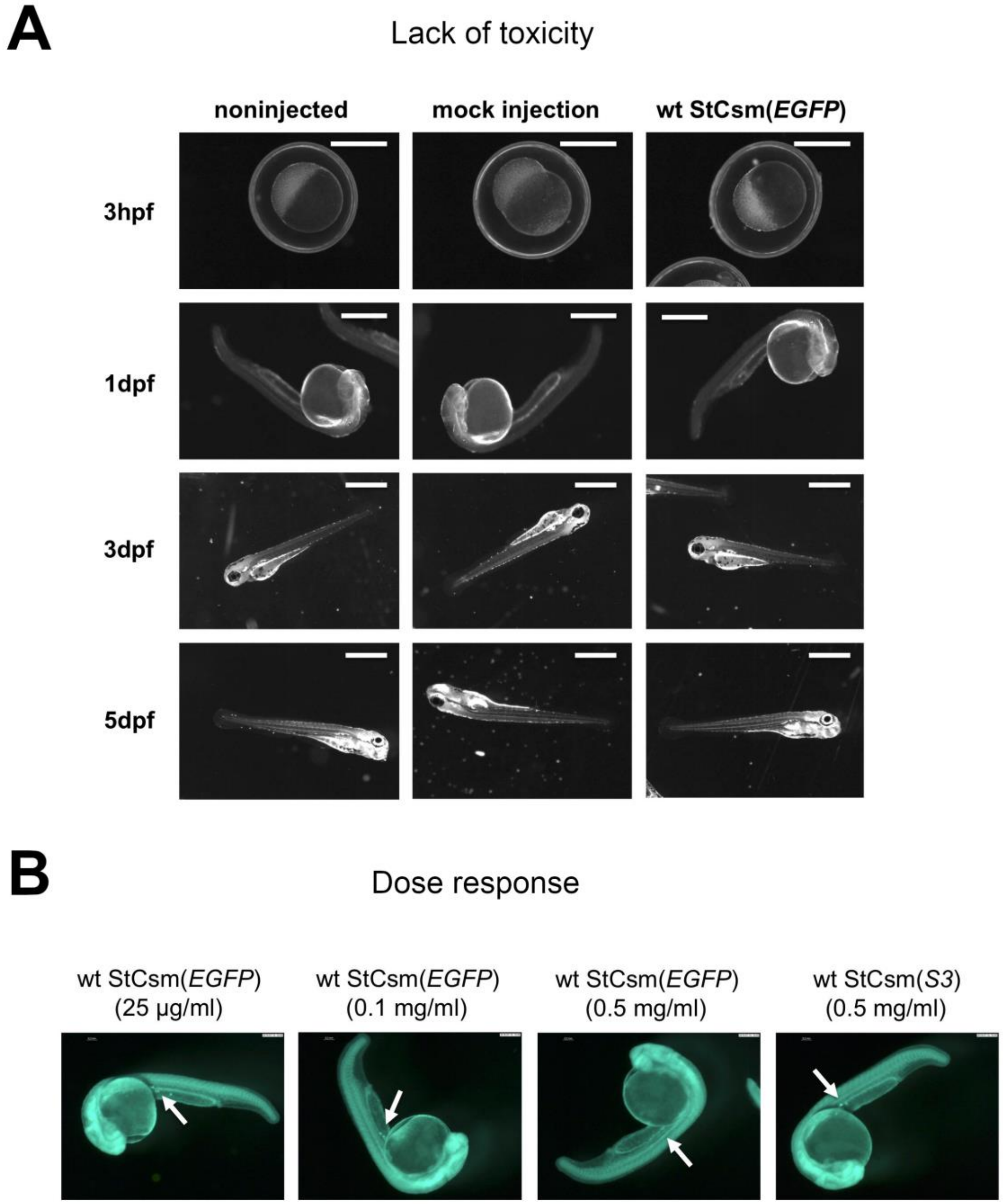

Supplement: Supplemental data [file Supp_Fig5.pdf]
