# Supplementary material for: Targeted RNA Knockdown by a Type III CRISPR-Cas Complex in Zebrafish
Source: CRISPR J. 2020 Aug 24;3(4):299–313. doi: 10.1089/crispr.2020.0032 (PMC7469701; doi:10.1089/crispr.2020.0032)

Figure S6: *Tg(Xla.Eef1a1:mlsEGFP)* in reciprocal crosses. Related to Figure 3. (A) *Tg(Xla.Eef1a1:mlsEGFP)* fluorescence was monitored in reciprocal crosses of ABTL and *Tg(Xla.Eef1a1:mlsEGFP)* parents. (B) Mean fluorescence was quantified 1dpf by FACS analysis of digested embryos. Results are from three independent experiments; error bars represent one standard deviation. *** P < 0.001 as compared with respective controls.

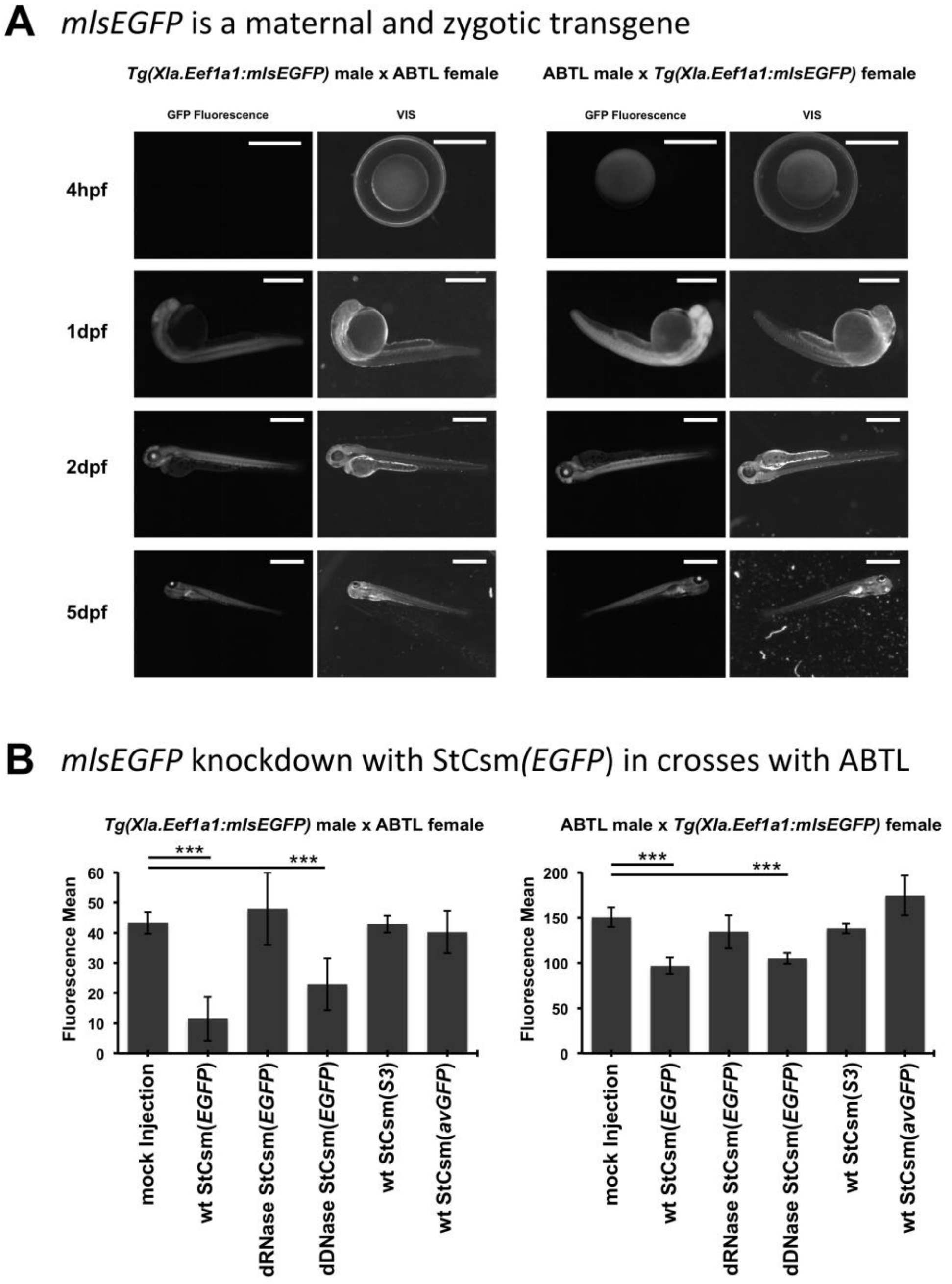

Supplement: Supplemental data [file Supp_Fig6.pdf]
