# Supplementary material for: Targeted RNA Knockdown by a Type III CRISPR-Cas Complex in Zebrafish
Source: CRISPR J. 2020 Aug 24;3(4):299–313. doi: 10.1089/crispr.2020.0032 (PMC7469701; doi:10.1089/crispr.2020.0032)

Figure S8: *Tg(fli1:EGFP)* knockdown. Related to Figure 3. The effect of the injection of StCsm crRNA complexes into *Tg(fli1:EGFP)* embryos was monitored.

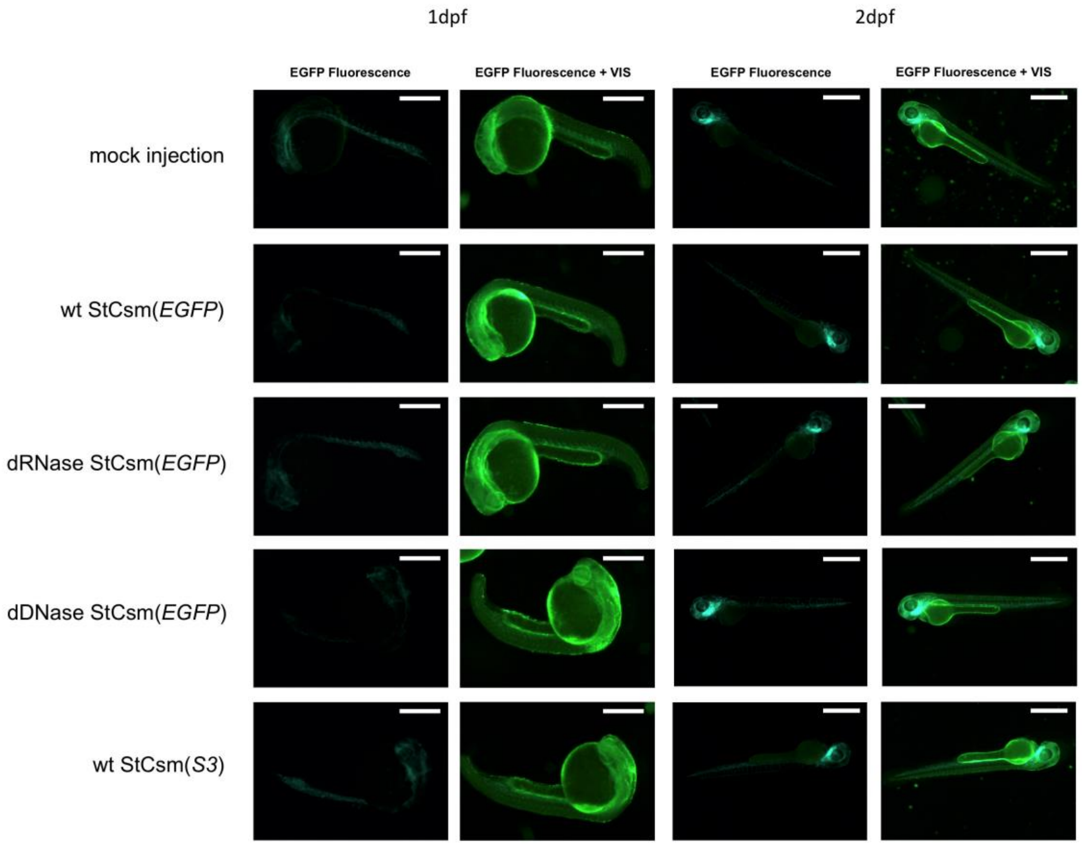

Supplement: Supplemental data [file Supp_Fig8.pdf]
