# Supplementary material for: Targeted RNA Knockdown by a Type III CRISPR-Cas Complex in Zebrafish
Source: CRISPR J. 2020 Aug 24;3(4):299–313. doi: 10.1089/crispr.2020.0032 (PMC7469701; doi:10.1089/crispr.2020.0032)

Figure S9: Microscopy of StCsm mediated *tdgf1* knockdown in wild type AB zebrafish. Related to Figure 4. Lateral and ventral images of embryos after 24hpf, post-injection with dDNase StCsm(*tdgf1*^{167,174,154,181}) complexes. The arrows indicate the eye or eyes of the fish. Injection was done at the 1-cell stage.

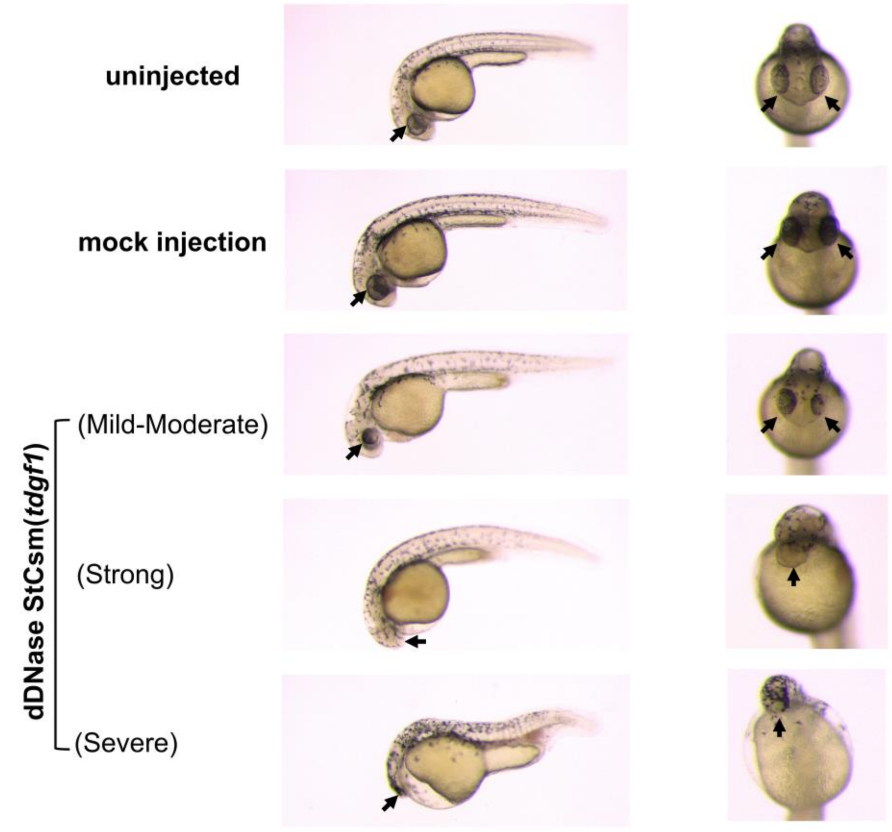

Supplement: Supplemental data [file Supp_Fig9.pdf]
