# Supplementary material for: Targeted RNA Knockdown by a Type III CRISPR-Cas Complex in Zebrafish
Source: CRISPR J. 2020 Aug 24;3(4):299–313. doi: 10.1089/crispr.2020.0032 (PMC7469701; doi:10.1089/crispr.2020.0032)

Figure S10. Read coverage analysis of the *EGFP* transcript at 128 cells stage. Related to Figure 5. The top two panels (StCsm 5hpf and Mock 5hpf) depict the read coverage represented as a fraction of total coverage at each position for the StCsm injected (red) and Mock injected (blue) samples. Each replicate is plotted independently with the color shades gaining intensity where the plots overlap. Third panel (LFC coverage) shows the log2 of the ratio between the read coverage of the StCsm injected and Mock injected samples (positive values red, negative values blue). The fourth and fifth panel (StCsm ends and Mock ends) depict the distribution of sequencing fragment ends over the whole *EGFP* transcript sequence for the StCsm injected (red) and Mock injected (blue) samples. The sixth panel (Diff. ends) shows the difference in the sequencing fragment end distribution between the two samples, obtained by subtracting the mean amount of fragment ends of the Mock sample from the mean amount of fragment ends at each coordinate of the StCsm sample. The positive and negative values are colored red and blue, respectively.

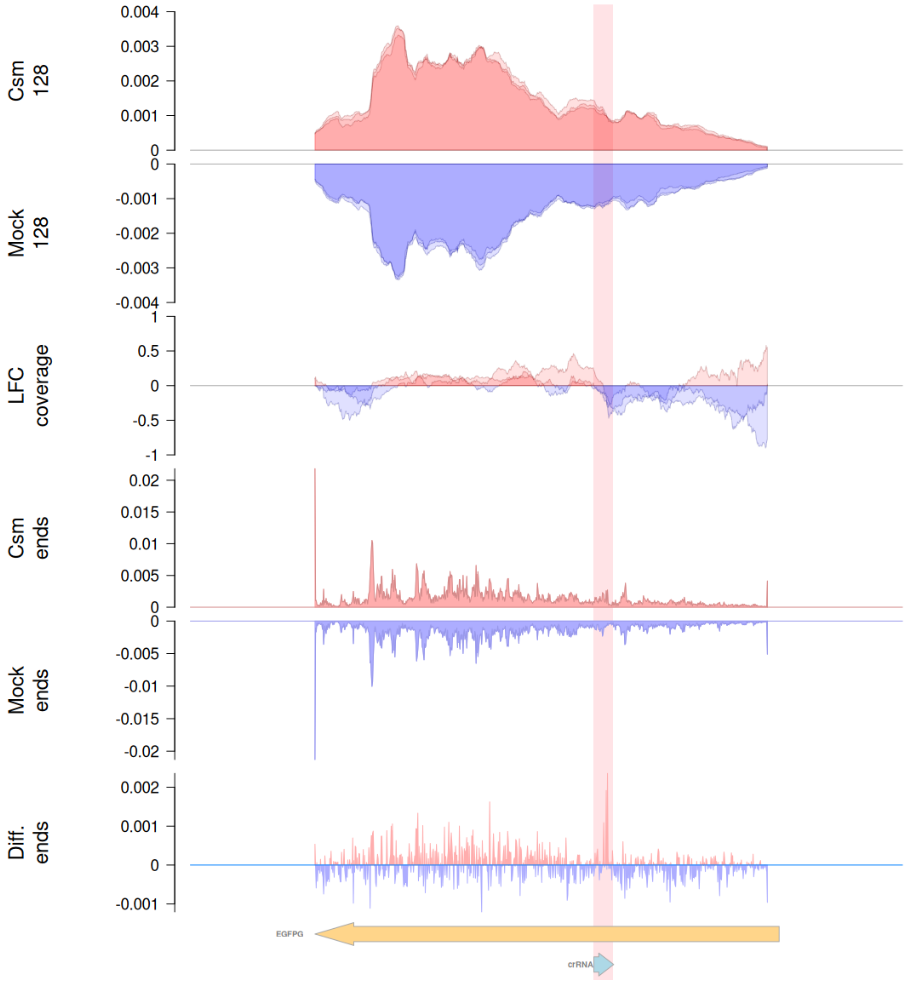

Supplement: Supplemental data [file Supp_Fig10.pdf]
