# Supplementary material for: Targeted RNA Knockdown by a Type III CRISPR-Cas Complex in Zebrafish
Source: CRISPR J. 2020 Aug 24;3(4):299–313. doi: 10.1089/crispr.2020.0032 (PMC7469701; doi:10.1089/crispr.2020.0032)

Figure S11: Effect of *EGFP* knockdown across the transcriptome. Related to Figure 6. (A) Correlation heatmap and hierarchical clustering of Euclidean sample-to sample distance show that developmental stage dominates the transcriptome, and that injection condition has only minor effects on the overall transcriptome. (B) MA plots of log2 fold changes of read counts over the mean of normalized counts for all tested time points. The plots have different y-axis scale. Every point in the scatterplot represents a gene. Genes with adjusted p-value lower than 0.1 are colored red. (C) Number of matching nucleotides is not a good predictor for off-target effect. The dependency of the Wald test statistic, as calculated by DESeq2 for the StCsm(*EGFP*) and Mock injected samples at 128 cell stage, on the number of complementary nucleotides to the crRNA. The hue of the points visualizes the log2 fold change between the experimental conditions. The higher the absolute value of the Wald test statistic is, the more statistically significant is the observed change.

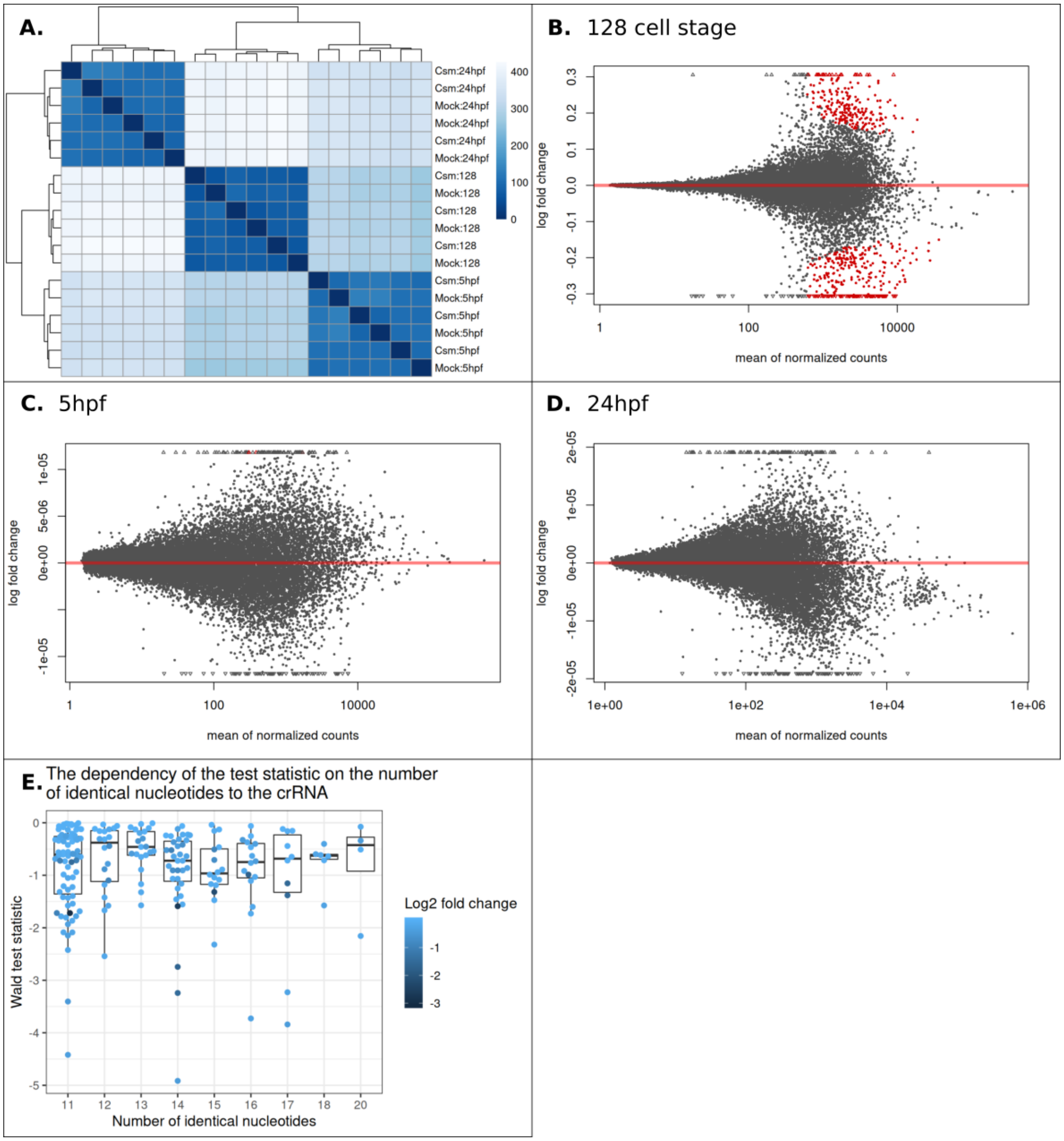

Supplement: Supplemental data [file Supp_Fig11.pdf]
